# *Origanum vulgare* ethanolic extracts as a promising source of compounds with antimicrobial, anti-biofilm, and anti-virulence activity against dental plaque bacteria

**DOI:** 10.3389/fmicb.2022.999839

**Published:** 2022-11-02

**Authors:** Fouzia Idir, Sybren Van Ginneken, Guglielmo A. Coppola, Daniel Grenier, Hans P. Steenackers, Farida Bendali

**Affiliations:** ^1^Laboratoire de Microbiologie Appliquée, Faculté des Sciences de la Nature et de la Vie, Université de Bejaia, Bejaia, Algeria; ^2^MiCA Lab, Centre of Microbial and Plant Genetics, KU Leuven, Leuven, Belgium; ^3^Oral Ecology Research Group, Faculty of Dentistry, Université Laval, Quebec, QC, Canada

**Keywords:** oral pathogenic bacteria, *Origanum vulgare* ethanolic extract, antimicrobial activity, anti-biofilm activity, thymol

## Abstract

Dental caries and periodontal diseases remain a challenge for oral health, especially given the lack of effective and safe treatment options that are currently available. Against the backdrop of an ongoing antimicrobial resistance crisis, a renewed interest in traditional medicinal plants as a potential source of new bioactive compounds has surfaced. In this context, we systematically screened the antimicrobial and anti-biofilm activities of both ethanolic and aqueous extracts of nine Algerian medicinal plants (*Artemisia herba alba, Centaurium erythraea, Juglans regia, Laurus nobilis, Matricaria recutita, Mentha pulegium, Mentha piperita, Origanum vulgare* and *Taraxacum officinale*). To evaluate the activity spectrum of the extracts, the screening was carried out against an extensive collection of *Streptococcus*, *Enterococcus* and *Lacticaseibacillus* isolates recovered from dental plaques of Algerian patients. Broad-spectrum antimicrobial and anti-biofilm properties were observed, especially among ethanolic extracts, which marks them as a promising source for bioactive compounds to control oral biofilms. The ethanolic extract of *O. vulgare*, which showed the most promising effects in the initial screening, was further characterized. We first verified the biocompatibility of this extract using human oral keratinocytes and selected a range of non-cytotoxic concentrations (0.195–0.781 mg/ml) to further validate its anti-biofilm and anti-virulence potential. At these concentrations, the extract not only prevented biofilm formation (10.04 ± 0.75–87.91 ± 9.08% of reduction) of most dental plaque isolates on a polystyrene surface, but also significantly reduced their adherence to hydroxyapatite (34.58 ± 9.09–62.77 ± 0.95%). Moreover, the extract showed curative potential against mature biofilms grown under conditions mimicking the oral niche. In addition to its anti-biofilm properties, we observed an inhibition of glucosyltransferase activity, a reduction in acidogenesis and a downregulation in the expression of multiple virulence-associated genes for extract-treated samples. Since anti-virulence properties are more robust to the development of resistance, they provide an attractive complementation to the antimicrobial activities of the extract. Thymol was identified as an important active compound of the extract using GC–MS analysis, but synergy with other compounds was also detected, suggesting a potential advantage of using the whole extract over purified thymol. Further research into the bioactive compounds of the *O. vulgare* ethanolic extract could yield novel products to fight dental caries.

## Introduction

Despite improvements in oral hygiene over the past decades, dental caries and periodontal disease remain the two most prevalent oral conditions worldwide, playing a major role in tooth decay and eventually tooth loss (Frencken et al., 2017). Dental caries is defined as a demineralization process of tooth enamel due to organic acids, mainly lactic acid, produced by oral bacteria that reside in dental plaque biofilm (Cugini et al., 2021). Dental plaques and caries are generally associated with periodontal disease, which indicates an infection of periodontal tissues such as the gingiva that leads to inflammation and eventually tissue destruction (Bunte et al., 2019). It has become clear that both conditions should be considered a consequence of ecologically driven imbalances of the microbiota in oral biofilms (Marsh, 1994). The oral microbiota can be affected by cultural and biological factors such as diet, environment, hygiene, physiology, health status, genetics, and lifestyle (Weyrich, 2021). Cariogenic dental plaque biofilms are typically associated with an overabundance of (mutans) streptococci and lactobacilli that are believed to play an active role in tooth decay (Tanzer et al., 2001). Nonetheless, the shift towards cariogenic oral biofilms can also involve other pathogens such as *Candida albicans* (Xiao et al., 2018). Regarding periodontal disease, mostly anaerobic Gram-negative bacteria have been implicated, including *Fusobacterium nucleatum*, *Aggregatibacter actinomycetemcomitans*, *Prevotella intermedia*, *Porphyromonas gingivalis*, or *Tannerella forsythia* (Sterzenbach et al., 2020). Especially *F. nucleatum* is believed to play an important role in the subgingival biofilm formation by bridging the early colonizers, mostly Gram-positive (e.g., streptococci), and the secondary colonizers, mostly Gram-negative (e.g. *P. gingivalis* and *T. forsythia*) (Kolenbrander et al., 2006). Enterococci have also been reported to play a role in oral infections, with *Enterococcus faecalis* being recognized as the main causal species of this group due to its ability to form refractory biofilms (Komiyama et al., 2016).

Given their high frequency and recalcitrance, prevention and treatment of oral infections receive considerable attention worldwide. Chlorhexidine is one of the most widely recognized antiseptics for oral hygiene and is used in the form of a varnish, mouthwash, or dentifrice gel; it has yielded beneficial results as a preventive strategy, and to improve the symptoms of periodontal disease (Oltramari-Navarro et al., 2009). However, despite its effectiveness in reducing the levels of microorganisms in the oral cavity, its long-term use is associated with local side effects such as impaired sense of taste, tooth staining, increased formation of supragingival calculus, and occasional irritation and desquamation of mucous membranes (Gürgan et al., 2006). Because of the toxicity of the currently used antimicrobials and the emergence of antimicrobial resistance, it is important to search for alternatives to control oral biofilms (Milho et al., 2021; Vaca et al., 2021). The search for safe, effective and economical compounds that treat or prevent oral diseases in order to outface the increasing bacterial resistance to currently used antimicrobials and chemotherapeutics has become necessary. For this purpose, medicinal plants have proven to be an abundant source of biologically active compounds, and have been the basis for the development of several new chemicals for pharmaceutical application (Palombo, 2011). Isolation and characterization of active molecules from medicinal plants which, can have an efficacy comparable to antibiotics but without exhibiting any adverse health effects, have gained the attention of researchers (Dadpe et al., 2018).

Numerous Algerian plants were traditionally used, for many decades, as remedies for various oral diseases without any major side effects. Unfortunately, few studies have been undertaken to prove their efficacy and to characterize their active compounds. To fill this gap and further tap into the potential of natural compounds, this study explored the antimicrobial properties of local medicinal plants against oral infections in a systematic and integrated way. First, an extensive collection of 83 oral isolates obtained from dental plaques of Algerian patients, comprising clinically relevant strains of *Lacticaseibacillus*, *Streptococcus* and *Enterococcus* was established. Using this clinical library, the antimicrobial and anti-biofilm properties of both aqueous and ethanolic extracts obtained from nine Algerian plants were systematically screened and compared to the activities observed against three reference strains (*Streptococcus mutans* ATCC 25175, *Candida albicans* ATCC 28366 and *Fusobacterium nucleatum* ATCC 25586). In addition, the anti-biofilm activity of the most promising extracts was further validated in more complex *in vitro* models using both hydroxyapatite and artificial saliva to mimic the oral niche. These assays were further complemented with an evaluation of the anti-virulence properties of the selected extracts, including their inhibition of glucosyltransferase activity, their anti-acidifying capacity, and their downregulation of various virulence-associated genes. Finally, the active compounds present in the extract showing the greatest potential for clinical application were further investigated using HPLC fractionation followed by GC–MS analysis.

## Materials and methods

### Plant collection

Nine local plants, traditionally used for treating oral diseases and dental pain in Algeria, were tested in this study. Herbalists in Bejaia (North-East Algeria) helped in identification and sampling of the plants. The names and the plant parts used are indicated in Table 1. The plants were collected, washed and dried in the dark at ambient temperature. Then, they were grinded using a blender (DAEWOO, France) until a fine powder was obtained.

**Table 1:** Plant name and part used in this study.

Common name in: Amazigh/English/ French	Scientific name	Part used	Code
Marzagu/Centaury/Petite centaurée	*Centaurium erythraea*	Aerial part	1
Wamlal/Chamomile/Camomille	*Matricaria recutita*	Flowers	2
Tuɣmest n temɣart/Dandelion/Pissenlit	*Taraxacum officinale*	Aerial part	3
Achih/Wormwood/Armoise	*Artemisia herba alba* L.	Leaves	4
Zaater/Oregano/Origan	*Origanum vulgare*	Leaves	5
Felgo/Pennyroyal/Menthe pouliot	*Mentha pulegium* L.	Leaves	6
Naanaa/Mint/Menthe	*Mentha piperita* L.	Aerial part	7
Agoussim/Walnut/Noyer	*Juglans regia* L.	Root bark	8
Arend/Laurel/Laurier	*Laurus nobilis* L.	Leaves	9

### Extraction

Two extraction methods were used: aqueous (maceration and infusion) and ethanolic extraction, using 50 g of each plant powder. For aqueous maceration, the powder was suspended in distilled water (250–300 ml), crushed vigorously for 5 min in a mortar and left to macerate for 48 h in the dark under gentle shaking (60 rpm). For aqueous infusion and ethanolic extraction, the powder was suspended in hot (100°C) distilled water for 15 min or 95% [v/v] ethanol (150–200 ml; Sigma-Aldrich, Steinheim, Germany) for 7 days, respectively. During the extraction, the suspensions were kept in the dark under gentle shaking. At the end of the above extraction steps, the extracts were centrifuged (4,000 g, 30 min, 4°C; Hettich Rotina 380R, Germany), filtered (Wattman paper n°1), evaporated using a rotavapor (Rotavapor R-114; BÜCHI, Flawil, Switzerland) and lyophilized until powders were obtained. All extracts were stored in the dark at 4°C.

### Bacterial strains and growth conditions

Strains of *Lacticaseibacillus*, *Streptococcus* and *Enterococcus* were isolated and purified as follows: Dental supragingival plaque samples were collected from 50 Algerian patients (aged between 17 to 72 years old) consulting different dental offices in Bejaia city. Written informed consent was obtained from each participant. The samples were tenfold serially diluted in a tryptone salt (TS) solution (0.9% [w/v] NaCl supplemented with 0.1% [w/v] tryptone; Sigma) and inoculated onto de Man, Rogosa and Sharpe (MRS) agar (Sigma; pH 5.4) and Mitis-Salivarius bacitracin agar (Sigma; pH 7). The plates were incubated at 37°C/48 h. After incubation, the colonies were sub-cultured on new plates and their purity was checked by Gram staining and catalase activity testing. Gram-positive and catalase-negative bacilli were stored in MRS broth (Sigma; pH 5.4) supplemented with 20% [v/v] glycerol (Sigma), whereas the Gram-positive and catalase-negative cocci regrouped in chains were stored in Brain Heart Infusion (BHI) broth (Sigma; pH 7) supplemented with 20% [v/v] glycerol.

Three laboratory reference strains of oral pathogens were included in this study: *Streptococcus mutans* ATCC 25175, *Candida albicans* ATCC 28366 and *Fusobacterium nucleatum* ATCC 25586. These strains were obtained from the American Type Culture Collection (ATCC; Manassas, VA, United States).

### Identification of clinical isolates by 16S rDNA sequencing

Total DNA of each clinical isolate was recovered from fresh cultures (18 h, 10^8^ CFU/ml) and purified using the DNeasy Blood and Tissue kit (QIAGEN, Germany) as recommended by the manufacturer for Gram-positive bacteria. A PCR was used for the amplification of the 16S rDNA gene using universal bacterial primers (F: 5’-AGAGTTTGATCCTGGCTCAG-3′, R: 5’-AAGGAGGTGATCCAGCCGCAG-3′). A mixture of 20 μl of PCR reagents and 5 μl of pure DNA was used for the PCR following the running program: initial denaturation of 5 min at 95°C, then 30 cycles of amplification (1 min of denaturation at 95°C, 30 s of annealing at 55°C and 1 min extension at 72°C), followed by a final extension cycle at 72°C during 10 min. The PCR products were separated on a 1% [w/v] agarose gel upon electrophoresis, carried out at 400 volt/1 h and DNA fragments corresponding to 1,500 bp were purified using a gel extraction Kit (GenElute^Tm^, Sigma-Aldrich, Spain). The 16S rDNA sequences were assembled into unique contigs with the BioEdit sequence alignment software (Hall, 1999) and analyzed using the NCBI-Standard Nucleotide BLAST^1^ (Altschul et al., 1990). For species identification of the isolates, only high percentage of identity (≥ 98%) with a single species was accepted (Barghouthi, 2011).

### Screening of isolated strains for their biofilm formation capacity

The ability of isolated clinical strains to form biofilms was tested using the Tissue Culture Plate (TCP) method as described by O’Toole and Kolter (1998) with slight modifications. Briefly, wells of sterile flat-bottom 96-well polystyrene microtiter plates were filled with 100 μl of Tryptic Soy Broth (TSB; Biokar Diagnostics, France) and 100 μl of a fresh bacterial suspension (18 h, 10^7^ CFU/ml) in TSB. After 2 h at 37°C, the bacterial suspensions were removed and the wells were washed twice with 200 μl of sterile TS solution. Then, sterile TSB was added and the plates were further incubated at 37°C/ 24 h. Wells containing 200 μl of sterile TSB were used as negative controls. The cultures were decanted and the wells were washed twice with sterile TS solution to remove non-adherent cells. Thereafter, adherent cells were fixed with 200 μl of absolute ethanol (Sigma) for 15 min, after which the ethanol was aspirated and the wells left to dry. Next, the fixed cells were stained with 200 μl of 0.1% [w/v] crystal violet (Biochem Chemopharma, Québec, Canada) for 20 min, and then washed with 200 μl of sterile TS solution for three times. Finally, the fixed stain was solubilized with 200 μl of 95% ethanol. Biofilm biomass was quantified by reading the absorbance (A) of each well at 595 nm using a Synergy MX multimode reader (BioTek Instruments, Winooski, VT, United States).

### Evaluation of the antimicrobial and anti-biofilm activities of the plant extracts

#### Agar disc diffusion method

All the extracts were filter-sterilized with 0.22 μm syringe filters (Fisher Scientific, France) and tested for their antimicrobial activity against 20 clinical isolates (bacilli and cocci) and three reference strains (*Str. mutans* ATCC 25175, *C. albicans* ATCC 28366 and *F. nucleatum* ATCC 25586) using the agar disc diffusion method. Hereto, fresh cultures (18 h, 10^7^ CFU/ml) of the target strains in TSB were inoculated on the surface of Tryptic Soy Agar (TSA; Biokar Diagnostics, France) using sterile swabs. Similarly, fresh cultures (18 h, 10^7^ CFU/ml) of *F. nucleatum* in Todd-Hewitt broth (THB; Becton Dickinson, Canada) supplemented with 0.001% hemin and 0.0001% vitamin K and *C. albicans* in Sabouraud broth (Biokar Diagnostics, France) were inoculated on the surface of THB agar supplemented with 0.001% hemin and 0.0001% vitamin K and Sabouraud agar, respectively. Afterwards, sterile paper discs charged with 50 μl of each filter-sterilized plant extract at a concentration of 50 mg/ml, prepared by dissolving the lyophilized plant extract powder in a mixture of distilled water and 5% [v/v] dimethyl sulfoxide (DMSO; Sigma), were deposited on the surface of the inoculated plates and the latter were incubated at 37°C/ 24 h aerobically, except *F. nucleatum* which was incubated anaerobically (80% N_2_, 10% CO_2_, and 10% H_2_) in an anaerobic chamber. After incubation, the plates were examined for the appearance of inhibition zones and their diameters were measured in mm.

#### Antimicrobial activity of plant extracts on planktonic cultures

The antimicrobial activities of all aqueous and ethanolic plant extracts against the growth of five *Streptococcus* and five *Lacticaseibacillus* clinical isolates (selected based on their strongest biofilm formation capacity), *Str. mutans* ATCC 25175 and *C. albicans* ATCC 28633 were assessed using a microtiter plate assay. The wells of 96-well polystyrene microtiter plates were filled with 100 μl of sterile TSB. Then, 50 μl of each plant extract (50 mg/ml), prepared as described above, were added. Finally, 50 μl of a fresh microbial suspension (18 h, 10^7^ CFU/ml) were added. Wells filled with only plant extract or microbial suspension were used as controls. The plates were incubated at 37°C/24 h. After incubation, the effect of the plant extracts on the microbial growth was determined by recording the optical density (OD) at 630 nm. The % of growth inhibition was calculated as follows:


$$\begin{array}{l} \%\mathrm{Growth inhibition}\\ =\frac{\left[\left({\mathrm{OD}}_{630\;\mathrm{nm}\;\mathrm{negative control}}-{\mathrm{OD}}_{630\;\mathrm{nm}\;\mathrm{experimental}}\right)\;x\;100\%\right]}{{\mathrm{OD}}_{630\;\mathrm{nm}\;\mathrm{negative control}}} \end{array}$$

The antimicrobial effect of the ethanolic *O. vulgare* extract fractions and pure thymol (CAS: 89–83-8) on dental plaque isolates was determined in an identical way using the OD at 595 nm. Each experiment was performed in triplicate using independently grown cultures.

#### Determination of The MIC and MBC values of plant extracts

The minimal inhibitory concentration (MIC) and minimal bactericidal concentration (MBC) values of the plant extracts were determined against eight clinical isolates (*Str. salivarius* SA3, *Str. mitis* SA5, *Str. vestibularis* S2P1, *Str. anginosus* S4.49, *Lb. paracasei* L2.2, *Lb. rhamnosus* L41.2, *En. durans* S1.41 and *En. faecalis* S2.43), using a broth micro-dilution method in 96-well microtiter plates. First, plant extracts were prepared at a concentration of 100 mg/ml in a mixture of sterile distilled water and 5% [v/v] DMSO, and filter-sterilized. Then, twofold serial dilutions of the extracts (final concentrations = 100–0.195 mg/ml) were prepared in 100 μl of TSB supplemented with 0.5% [w/v] glucose (Sigma) in 96-well polystyrene microtiter plates. Afterwards, 100 μl of fresh bacterial suspensions (18 h, 10^7^ CFU/ml), prepared in sterile TSB supplemented with 0.5% [w/v] glucose, were added to each well. The microtiter plates were incubated for 24 h at 37°C. Control wells without bacterial suspension or plant extract were included. Bacterial growth was monitored by reading the OD_630nm_. The MIC values were the lowest concentrations of the plant extracts where the bacterial growth was totally inhibited. To determine the MBC values, aliquots of 10 μl were aseptically recovered from the wells in which no visible growth was registered, and plated on TSB agar plates after which these plates were incubated for 48 h at 37°C. The MBC values were the lowest concentrations where no growth of colonies was observed. Each experiment was performed in triplicate using independently grown cultures.

#### Anti-biofilm activity of plant extracts

Wells of a flat-bottom 96-well microtiter plate, filled with 100 μl of sterile TSB and 50 μl of each plant extract (50 mg/ml), were prepared as described above. Then, 50 μl of fresh microbial culture (18 h, 10^7^ CFU/ml) of the same strains used in section 2.6.2 were added. After 2 h at 37°C, the microbial suspensions were removed and the wells were washed twice with 200 μl of sterile TS solution. Then, sterile TSB was added and the plates were re-incubated at 37°C/24 h. Wells, filled with only plant extract or microbial suspension, were used as controls. The evaluation of the anti-biofilm activity of the plant extracts was carried out as previously described in the section 2.4 (O’Toole and Kolter, 1998). The % biofilm inhibition was calculated as follows:


$$\begin{array}{l} \%\mathrm{Biofilm inhibition}\\ =\frac{\left[\left(A_{595\;\mathrm{nm}\;\mathrm{negative control}}-A_{595\;\mathrm{nm}\;\mathrm{experimental}}\right)\;x\;100\%\right]}{A_{595\;\mathrm{nm}\;\mathrm{negative control}}} \end{array}$$

Similar biofilm experiments in which plant extracts were mixed with microbial cultures and incubated for 24 h at 37°C were performed with different concentrations (1.56, 0.781 and 0.390 mg/ml) of the *O. vulgare* ethanolic extract. The anti-biofilm activity of the extract fractions and pure thymol (CAS: 89–83-8) on dental plaque isolates was determined in an identical way. Each experiment was performed in triplicate using independently grown cultures.

### *In vitro* biocompatibility

The human oral keratinocyte cell line B11 (Gröger et al., 2008), which was kindly provided by S. Gröger (Justus Liebig University Giessen, Germany), was cultured in keratinocyte serum-free medium (K-SFM; Life Technologies Inc., Burlington, ON, Canada) supplemented with growth factors (50 μg/ml of bovine pituitary extract and 5 ng/ml of human epidermal growth factor) and 100 μg/ml of the penicillin G-streptomycin solution. The cells were seeded (1 *×* 10^5^ cells in 100 μl) in the wells of a 96-well microplate (100 μl/well) and were cultured overnight at 37°C in a 5% CO_2_ atmosphere until they reached confluence. The keratinocytes were treated for 5 min or 120 min with the extracts (50 ng/ml - 50 mg/ml). The wells were then immediately washed with Dulbeco’s Modified Eagle Medium (DMEM; Life Technologies Inc., Burlington, ON, Canada) prior to assessing cell viability using a MTT (3-[4,5-diethylthiazol-2-yl]-2,5-diphenyltetrazolium bromide) assay, performed according to the manufacturer’s protocol (Roche Diagnostics, Mannheim, Germany). Untreated cells were used to establish 100% viability (negative control). The assays were carried out in triplicate, and the means ± standard deviations (SD) were calculated.

### Inhibitory effect of the *Origanum vulgare* ethanolic extract on adherence to hydroxyapatite-coated surfaces

First, white-bottom microplates coated with hydroxyapatite were prepared as previously described by Shahzad et al. (2015). Briefly, a hydroxyapatite suspension was prepared at 10% [w/v] in acetone (Sigma). Afterwards, 60 μl aliquots of the suspension were transferred into 96-well white-bottom microplates (Thermo Fisher Scientific, Finland). The latter were incubated with shaking (200 rpm) at 37°C/15 min. Then, non-adherent hydroxyapatite was removed by inverting the microplates and the wells were washed twice with Phosphate Buffered Saline (PBS; pH 7.4), prepared using 8.8 g/l NaCl, 1.24 g/l K_2_HPO_4_ and 0.39 g/l KH_2_PO_4_, and dried. The microplates were sterilized with UV using an UV visible light lamp. Volumes of 10 ml of fresh bacterial cultures (18 h, 10^7^ CFU/ml) of the eight clinical isolates (see section 2.6.3) grown in TSB were centrifuged (10,000 g/5 min, 4°C), and the pellets were washed twice with PBS and suspended in 10 ml of fresh sodium bicarbonate buffer (0.5 M, pH 8). Afterwards, 30 μl of fluorescein isothiocyanate (FITC) solution prepared at 10 mg/ml in 95% ethanol was added to the cultures, followed by incubation at 37°C/60 min in the dark. Following incubation, the bacterial suspensions were washed three times with PBS and diluted in the same buffer to obtain an OD_630nm_ of 1.0. Equal volumes (100 μl) of the bacterial suspension and the plant extract (1.56, 0.781 or 0.390 mg/ml) were added into wells of the microplates and incubation was carried out at 37°C/2 h. Thereafter, the wells were washed twice with PBS and the effect of the extract on the adherence of the strains was evaluated by monitoring relative fluorescence (RF) using a Synergy MX multimode reader (excitation wavelength of 495 nm, and emission wavelength of 525 nm). The % inhibition of adherence to hydroxyapatite-coated surfaces was calculated as follows:


$$\begin{array}{l} \%\mathrm{Inhibition of adherence}\\ =\frac{\left[\left({\mathrm{RF}}_{\mathrm{negative control}}-{\mathrm{RF}}_{\mathrm{experimental}}\right)\;x\;100\right]}{{\mathrm{RF}}_{\mathrm{negative control}}} \end{array}$$

Each experiment was performed in triplicate using independently grown cultures.

### Biofilm eradication capacity (polystyrene surface) of the *Origanum vulgare* ethanolic extract

Artificial saliva was prepared by mixing porcine stomach mucin (0.25%, [w/v]), sodium chloride (0.35%, [w/v]), potassium chloride (0.02%, [w/v]), calcium chloride dihydrate (0.02%, [w/v]), yeast extract (0.2%, [w/v]), Lab-Lemco powder (0.1%, [w/v]) and proteose peptone (0.5%, [w/v]) into 1 l of distilled water. After autoclaving, sterile urea was added at 0.05% [v/v]. The final pH of the prepared saliva was adjusted to pH 7.4 (Shahzad et al., 2015). A volume of 100 μl of a fresh culture (18 h, 10^7^ CFU/ml) of each clinical isolate (see section 2.6.3) was added to 100 μl of sterile TSB or artificial saliva into wells of a 96-well microplate, which was incubated at 37°C/ 24 h. Similarly, 7-day biofilms were prepared into wells of 96-well microplates. Hereto, 100 μl of a fresh culture (18 h, 10^7^ CFU/ml) of each clinical isolate in TSB was added to 100 μl of sterile TSB or artificial saliva, and incubation was carried out for 7 days at 37°C. Every 2 days, bacterial suspensions were removed and replaced by sterile TSB or artificial saliva. Following incubation, the pre-formed biofilms (24-h and 7-day old) were treated with twofold serial dilutions of *O. vulgare* ethanolic extract (12.5–0.195 mg/ml) prepared in TSB or artificial saliva and then incubated for 2 h. Pre-formed biofilms (24-h and 7-day old) treated with only TSB or artificial saliva were included in the microplates as negative controls. Next, the bacterial suspensions were removed and the biofilms were washed twice with PBS. Finally, the detachment of the biofilm was performed with sonication (45,000 Hz, 10 min) using a water bath sonicator (VWR USC 300-T) and the number of viable cells contained in the biofilm was counted by plating on TSA and expressed as CFU/mL.

### Biofilm eradication capacity (hydroxyapatite surface) of the *Origanum vulgare* ethanolic extract

A cell viability assay based on the reduction of resazurin by metabolically active cells was used to evaluate the effects of *O. vulgare* ethanolic extract on eradication of 24-h and 7-day old biofilms by the method described by Sandberg et al. (2009). First, 24-h and 7-day old biofilms of selected clinical isolates (*Str. mitis* SA5, *Lb. paracasei* L2.2 and *En. faecalis* S2.43) were pre-formed using TSB or artificial saliva, into hydroxyapatite-coated wells of white-bottom microplates, and treated with *O. vulgare* ethanolic extract (12.5–0.195 mg/ml) as previously described in section 2.9. Pre-formed biofilms (24-h and 7-day old) treated with only TSB or artificial saliva were included as negative controls. Following incubation (37°C/2 h), wells were washed twice with PBS and filled with a sterile resazurin solution, prepared in TSB at 10% [w/v], and re-incubated for 2 h. Afterwards, the resazurin solution was transferred into sterile 96-well polystyrene microplates and the relative fluorescence (RF) was measured (excitation wavelength: 540 nm, emission wavelength: 590 nm). The % of biofilm eradication was calculated as follows:


$$\begin{array}{l} \%\mathrm{Biofilm eradication}\\ =\frac{\left[\left({\mathrm{RF}}_{\mathrm{negative control}}-{\mathrm{RF}}_{\mathrm{experimental}}\right)\;x\;100\right]}{{\mathrm{RF}}_{\mathrm{negative control}}} \end{array}$$

Each experiment was performed in triplicate using independently grown cultures.

### Inhibitory effect of the *Origanum vulgare* ethanolic extract on the biosynthesis of water insoluble glucans

The effect of the *O. vulgare* ethanolic extract on the biosynthesis of water insoluble glucans by eight clinical isolates (see section 2.6.3) was investigated. First, crude free glucosyltransferase was recovered as previously described (Furiga et al., 2008) with few modifications. Briefly, a fresh culture of each isolate (18 h, 10^7^ CFU/ml) in fresh TSB, supplemented with 0.5% [w/v] glucose, was centrifuged (13,500 g/30 min, 4°C) and filter-sterilized using a 0.45 μm membrane filter. Afterwards, the proteins contained in the supernatant were precipitated by adding ammonium sulfate (50% of saturation) under agitation for 30 min at 4°C. After centrifugation (12,000 g/30 min, 4°C), the precipitates were dissolved in potassium phosphate buffer (0.0625 M, pH 6.5). To determine the inhibitory effects, 25 μl of crude free glucosyltransferase and 175 μl of plant extract, prepared as described above to obtain final concentrations of 0.781, 0.390 and 0.195 mg/ml, were added to 0.8 ml of potassium phosphate buffer (0.0625 M, pH 6.5) supplemented with 12.5 mg/l of sucrose and 0.25 mg/l of sodium azide (Sigma). Assays, in which the plant extract was replaced by 5% [v/v] of DMSO in sterile distilled water, were considered as negative controls (100% synthesis). The mixtures were then incubated at 37°C/2 h prior quantifying the insoluble glucan synthetized by recording the absorbance at 550 nm. The inhibitory effect on glucosyltransferase activity was calculated as follows:


$$\begin{array}{l} \%\mathrm{Inhibition}\\ =\frac{\left[\left(A_{550\;\mathrm{nm}\;\mathrm{negative control}}-A_{550\;\mathrm{nm}\;\mathrm{experimental}}\right)\;x\;100\right]}{A_{550\;\mathrm{nm}\;\mathrm{negative control}}} \end{array}$$

Each experiment was performed in triplicate using independently grown cultures.

### Inhibitory effect of the *Origanum vulgare* ethanolic extract on the acidifying activity

The effect of the *O. vulgare* ethanolic extract on the acidifying activity of clinical isolates was determined as previously described by Shafiei et al. (2020). Fresh overnight cultures (18 h, 10^7^ CFU/ml) of the tested isolates (see section 2.6.3) were centrifuged (13,500 g/30 min, 4°C). The pellets were washed twice with saline solution (pH 7.0) prepared with 50 mM KCl and 1 mM MgCl_2_, and suspended in the same saline solution. A volume of 25 ml of the bacterial suspension and 25 ml of the plant extract diluted in the same saline solution were mixed. The final concentrations of the plant extracts in the mixtures were 0.781 mg/ml, 0.390 mg/ml and 0.195 mg/ml, respectively. The pH of the mixtures was adjusted with 0.2 M KOH to a final value of 7.2 and glucose was added to the mixtures to a final concentration of 1% [w/v]. After 2 h of incubation at 37°C, the pH of the mixtures was measured with a pH meter (InoLab pH 7,110; WTW, Weilheim, Germany). Each experiment was performed in triplicate using independently grown cultures.

### Inhibition of virulence gene expression by the *Origanum vulgare* ethanolic extract

Total RNA prepared from bacterial biofilms, treated with *O. vulgare* ethanolic extract or DMSO as a control, was recovered using the SV total RNA isolation System (Promega, USA) following the instructions of the manufacturer. Recovered RNA was treated with DNase (1 unit/2 μg RNA) and the purity of the RNA preparation was assessed by electrophoresis on an agarose gel (1%, [w/v]). An aliquot of 500 ng of each RNA sample was used for the synthesis of relative cDNA using a cDNA synthesis kit (Thermo Scientific) according to the manufacturer’s protocol. A volume of 2 μl of diluted cDNA samples was added to the final volume of 18 μl of the reaction mixture containing: 10 μl of SYBR Green (Power SYBR Green or Bioline), 0.9 μl of 2 μM target gene primer Forward, 0.9 μl of 2 μM target gene primer Reverse (Table 2) and 3.2 μl of nuclease free water. Real-time quantitative PCR (RT-qPCR) was performed using a StepOneTM Real-Time PCR System (Applied Biosystems) and the SYBR Green was used as a program of run. Real-time PCR cycle parameters included 10 min at 95°C followed by 40 cycles involving denaturation at 95°C for 15 s, annealing at 60°C for 20 s and elongation at 72°C for 20 s. All the real-time PCR experiments were performed in triplicate and data expressed as the mean of at least three independent experiments. The expression levels of the target genes were normalized using the 16S rRNA gene for each strain and evaluated by relative quantification values calculated with the 2-ΔΔϹt method (Hellemans et al., 2007).

**Table 2:** Primers used in the RT-qPCR experiment.

Bacterial strain	Protein (*gene*)	Primers	References
*Str. mitis*	Biofilm-associated protein (*bapA1*)	F: 5′-ACG GCA GAT CCA GAT TCA AC-3′ R: 5′-CAG TGC TCA AGC TAC CGTC A-3’	Khan et al. (2016)
	Glycosyltransferase B (*gtfB*)	F: 5′-AAA ATG GCA CCC ACT TTC TG-3′ R: 5′-TTC TCT CAT CCG TTC TGG CT-3’	
	16S Ribosomal RNA (16S rRNA)	F: 5′-CRC CTG GGG AGT RCR G-3′ R: 5′-AGG GTT GCG CTC GTT G-3’	
*En. faecalis*	Gelatinase *(gelE)*	F: 5′-GGG GCA ATA CAG GGA AAA AT-3′ R: 5′-TCC TTC CCC AGT TTC CTT TT-3’	Lee and Tan (2015)
	Cytolysin activator (*cylA*)	F: 5′-GAC TCG GGG ATT GAT AGG C-3′ R: 5′-TTT CCA TCT GTC CCA TCC AT-3’	
	Serine protease (*sprE*)	F: 5′-GCG TCA ATC GGA AGA ATC AT-3′ R: 5′-CAT CTT TGG CAT TCG GAT TT-3’	
	Collagen adhesin of *Enteroccocus* (*aceE*)	F: 5′-GGA ATG ACC GAG AAC GAT GGC T-3′ R: 5′-ATT CGG TTG CGA ACT ATT GG-3’	
	16S ribosomal RNA (16S rRNA)	F: 5’-CCG AGT GCT TGC ACT CAA TTG G − 3′ R: 5’-CCT TGG TGA GCC GTT ACC T-3’	
*Lb. paracasei*	Pyruvate oxidase (*spxB*)	F: 5′-GTG CCG ACG TTA TTT CTT G-3′ R: 5′-ATC ACA ACA ATC GCA GCT C-3’	Reis et al. (2021)
	Pilin protein (*spaC*)	F: 5′-GGT CAG GGA GAA GCG TAC T-3′ R: 5′-CGG TGT GAC GAC TTA CCA T-3’	
	16S ribosomal RNA (16S rRNA)	F: 5′-GTG CTT GCA CCG AGA TTC AAC ATG-3′ R: 5′-TGC GGT TCT TGG ATC TAT GCG-3’	

### Fractionation of the *Origanum vulgare* ethanolic extract and purification

The isolation of active compounds of the *O. vulgare* ethanolic extract was carried out in two steps. First, fractions with different polarity were isolated through column chromatography. To this end, the ethanolic extract was dried and adsorbed on a silica gel (Acros Organics, silica gel for chromatography, 0.060–0.200 mm, 60 A). The silica slurry was charged onto a column packed with heptane. The eluent polarity was increased in a stepwise manner: heptane (100%), heptane-ethylacetate (50:50%), dichloromethane-methanol (60:40%) and methanol (100%). The fractions showing biological activities were further purified by preparative high pressure liquid chromatography (Prep HPLC Column: VDS optilab VDSpher 100 SIL 5 μm, 20.0 × 30 mm) in a gradient (100% heptane ➔ 100% ethylacetate) coupled with an automated fractionating system (CombiFlash® EZ Prep, Teledyne ISCO).

### Characterization of the active fractions with GC–MS analysis

The purified fractions were characterized *via* a combination of gas chromatography and mass spectrometry (GC–MS). The gas chromatographer (Thermo Finnigan Trace GC Ultra 2.2) was equipped with a GC capillary column (Restek Rxi-5 ms 30 m, 0.25 mm ID, 0.25 μm df) and coupled to a mass spectrometer (Thermo Fisher ITQ 900 2.2, with an electron ionizer [EI] and an ion trap mass analyzer) using an auto sampler unit (Thermo Quest AS 2000). The system was set at an oven temperature of 300°C and a ramp rate of 20°C/min; split/splitless inlet (SSL) injector temperature of 250°C, split flow 60 ml/min and split ratio 50; MS transfer line at 300°C. The resulting spectra were analyzed with the NIST Mass Spectral Search 2.0 program for compound identification through a library matching.

### Statistical analysis

The comparison of the biofilm formation capacity of the clinical isolates (section 2.5) was performed with a student’s *t*-test. The results obtained in the sections 2.6, 2.8, 2.9, 2.10, 2.11, 2.12, 2.13 and 2.13 were analysed using two-way ANOVA (*p* < 0.1) and the comparison between the treated samples and the negative controls was performed with the Dunnett’s multiple comparisons test, whereas the comparison between the calculated percentages was performed with Tukey’s multiple comparison test. All statistical analyses was performed using GraphPad Prism 8.3.2.

## Results

### Screening of the bacterial isolates for their biofilm formation potential and their molecular identification

To establish an extensive collection of relevant oral bacteria for use during subsequent assays, 83 clinical isolates were recovered from dental plaques of Algerian patients. Of these, 53 were cocci and 30 were bacilli. All isolates were found to be Gram-positive and catalase-negative. From this collection, 40 isolates (20 bacilli and 20 cocci) were selected based on their strong ability to form biofilms (Supplementary Figure S1, Supplementary material), which makes them highly relevant targets in the context of dental caries and periodontal disease. In general, a *t*-test demonstrated a higher ability to form biofilms by the cocci compared to the bacilli. The 40 selected isolates were identified using 16S rDNA sequencing (Supplementary Table S1, Supplementary material). Among the 20 bacilli, 16 were identified as *Lacticaseibacillus rhamnosus* (L2, L11, L14, L15, L16, L17, L21, L27, L33, L36, L41.1, L41.2, L46, L50, L51 and L55) and four as *Lacticaseibacillus paracasei* (L2.2, L2.22, L2.26 and L6). The 20 cocci were identified as *Streptococcus salivarius* (SA3), *Streptococcus mitis* (S2.44, S1.43, SA5, S1.22, SA2 and S1.49), *Streptococcus anginosus* (S4.1 and S4.49), *Streptococcus vestibularis* (S2P1), *Enterococcus durans* (S1.41) and *Enterococcus faecalis* (S2P2, S3P2, S2.43, S4.46, S5.49, SF1, S1.33, S1.72b and S1.63b).

### Screening of plant extracts for their antimicrobial and anti-biofilm activities

In search of novel antimicrobials to combat oral infections and improve oral health, 27 extracts from Algerian plants with a history of traditional medicinal use were obtained following three extraction methods: ethanolic extraction, aqueous extraction by infusion, and aqueous extraction by maceration. The ethanolic extracts were codified E1-9, the aqueous extracts obtained by infusion were codified I1-9 and those obtained by maceration were codified M1-9, where 1–9 represents the number attributed to the plant (Table 1). To characterize the therapeutical potential of these plants, the obtained extracts were systematically screened for antimicrobial and anti-biofilm activities against the established library of dental plaque isolates.

### Screening the antimicrobial activity of the plant extracts following an agar disc diffusion approach

**Figure 1:**
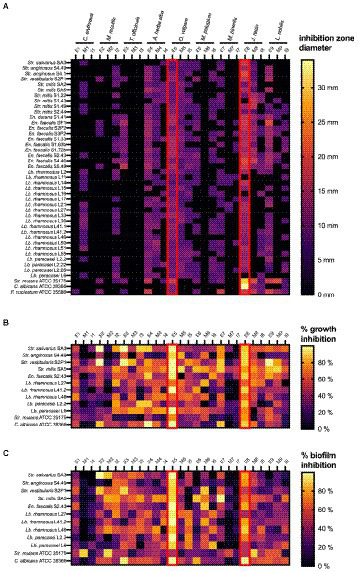
Heat maps representing the systematic screening of the antimicrobial activity of 27 plant extracts against a collection of dental plaque isolates and laboratory reference strains. **(A)** Growth inhibition observed in an agar disc diffusion assay, represented as inhibition zone diameters (mm). **(B)** Planktonic growth inhibition (%) compared to a non-treated negative control. **(C)** Biofilm growth inhibition (%) compared to a non-treated negative control. Results were obtained as the mean of three independent replicates and can be found in Supplementary Tables S2–S5 (Supplementary material). E, ethanolic extract; I, aqueous extract obtained by infusion; M, aqueous extract obtained by maceration.

As a first step in our screening approach, the antimicrobial activity of the plant extracts was evaluated using an agar disc diffusion assay against the 40 clinically relevant and strong biofilm producing dental plaque isolates (20 *Lacticaseibacillus*, 10 *Streptococcus* and 10 *Enterococcu*s). In addition, three laboratory strains were included as reference (*Str. mutans* ATCC 25175, *C. albicans* ATCC 28366 and *F. nucleatum* ATCC 25586). The results of this systematic screening are summarized in Supplementary Tables S2, S3 (Supplementary material) and presented as a heat map in Figure 1A. Overall, the largest zones of growth inhibition (up to 34 mm) were observed for the ethanolic extract of *Juglans regia* (E8). Most extracts showed broad-range antimicrobial activity against multiple *Lacticaseibacillus*, *Streptococcus* and *Enterococcus* isolates. Especially the *Origanum vulgare* E5 and M5 extracts showed a remarkable spectrum of activity, with zones of growth inhibition observed against all 40 oral isolates included in the assay. In contrast, the *Centaurium erythraea* E1 and I1, *Matricaria recutita* M2, *Taraxacum officinale* M3 and I3, and *Mentha pulegium* M6 extracts did not show antibacterial activity against any of the 40 isolates tested. Interestingly, some extracts showed a narrower activity spectrum. For example, the *J. regia* M8 extract showed activity against all included *Streptococcus* and *Enterococcus* isolates, but not against any of the *Lacticaseibacillus* isolates. In a similar fashion, the *T. officinale* E3 extract inhibited the growth of all *Enterococcus* isolates, but had no effect on the growth of *Streptococcus* or *Lacticaseibacillus* isolates (with the exception of *Str. vestibularis* S2P1).

#### Screening the antimicrobial activity of the plant extracts on planktonic cultures

Based on their strong biofilm forming capacity, 10 clinical isolates (4 *Lacticaseibacillus*, 5 *Streptococcus* and 1 *Enterococcus*) and two reference strains (*Str. mutans* ATCC 25175 and *C. albicans* ATCC 28366) were selected to further investigate the antimicrobial properties of the plant extracts. The percentages of planktonic growth inhibition exerted by 12.5 mg/ml of the plant extracts are shown in Supplementary Table S4 (Supplementary material) and presented as a heat map in Figure 1B. In general, all plant extracts tested were found to exert at least some antimicrobial activity against most of the 12 microorganisms tested. Interestingly, this is true even for the extracts that showed little to no antimicrobial activity in the agar disc diffusion assay (Figure 1A). In agreement with the agar disc diffusion assay, high percentages of growth inhibition across a broad spectrum of clinical isolates were observed with the ethanolic extracts of *O. vulgare* (E5; 50–99.59%) and *J. regia* (E8; 64.25–90.08%).

#### Screening the anti-biofilm activity of the plant extracts

In addition to the inhibitory effect on planktonic growth, the ability of the plant extracts to inhibit biofilm formation was evaluated using the same panel of 10 clinical isolates and two reference strains. The results are shown in Supplementary Table S5 (Supplementary material) and are presented as a heat map in Figure 1C. Overall, most extracts demonstrated anti-biofilm potential against multiple of the tested microorganisms. Specifically for the ethanolic extract of *O. vulgare* (E5), remarkable percentages of biofilm inhibition (46.33–99.84%) were observed against all 12 tested isolates. In contrast, little to no anti-biofilm activity was observed with the *C. erythraea* M1 and I1, and the *M. piperita* M7 and I7 extracts. Interestingly, some extracts, for example the *M. recutita* M2 extract, consistently demonstrated a more pronounced anti-biofilm effect compared to their planktonic growth inhibition, suggesting a mode-of-action specifically targeting biofilm formation. Taken together, the data presented in Figure 1 puts forward the ethanolic extracts of *O. vulgare* (E5) and *J. regia* (E8) as the most promising plant extracts with strong antimicrobial and anti-biofilm capacities against a broad spectrum of oral pathogens.

#### MIC and MBC determination of the plant extracts

To further quantify the range of extract concentrations that effectively inhibit microbial growth, MIC and MBC assays were carried out for all 27 extracts against a subset of eight clinical isolates with a strong biofilm forming capacity. Interestingly, even at the highest concentration tested (100 mg/ml), no complete growth inhibition could be observed for any of the 18 aqueous extracts, suggesting that both the MIC and MBC values exceeded 100 mg/ml. On the contrary, all ethanolic extracts completely inhibited the growth of at least one target microorganism in the tested concentration range, allowing the determination of a MIC value (Table 3). Only the ethanolic extracts of *O. vulgare* (E5) and *J. regia* (E8) had a MIC value <100 mg/ml against all tested oral microorganisms, with MICs ranging from 1.56 to 25.0 mg/ml (*O. vulgare*) and from 12.5 to 25.0 mg/ml (*J. regia*). Of the ethanolic extracts, the least effective one was the *C. erythraea* extract (E1), which had a detectable MIC only against *Str. salivarius* SA3 (25 mg/ml). In general, the MBC values tended to be higher compared to the MIC values, indicating that higher extract concentrations are required to obtain a bactericidal effect. The most bactericidal extract was the *O. vulgare* ethanolic extract (E5) with MBCs ranging between 3.125 and 25 mg/ml. In summary, the *O. vulgare* ethanolic extract (E5) showed the strongest antimicrobial activity against a broad spectrum of oral pathogens in both the MIC and MBC assays. Taken together with the promising antimicrobial and anti-biofilm activity of the E5 extract also observed in the previous screening assays (Figure 1), this extract was selected for further characterization.

**Table 3:** MIC/MBC values (mg/mL) of ethanolic plant extracts (E1-9) against selected oral isolates.

Bacterial isolate	Ethanolic plant extracts (E1-9)								
	*Centaurium erythraea* (E1)	*Matricaria recutita* (E2)	*Taraxacum officinale* (E3)	*Artemisia herba alba* (E4)	*Origanum vulgare* (E5)	*Mentha pulegium* (E6)	*Mentha piperita* (E7)	*Juglans regia* (E8)	*Laurus nobilis* (E9)
*Str. salivarius* SA3	25/50	1.563/6.25	3.125/6.25	12.5/25	**1.563/3.125**	25/25	0.781/1.563	12.5/25	12.5/25
*Str. mitis* SA5	> 100	25/25	25/50	> 100	**12.5/12.5**	12.5/25	> 100	12.5/25	25/25
*Str. vestibularis* S2P1	> 100	3.125/6.25	6.25/12.5	25/25	**6.25/6.25**	25/ 25	12.5/12.5	12.5/25	3.125/6.25
*Str. anginosus* S4.49	> 100	25/50	25/50	> 100	**12.5/12.5**	12.5/25	> 100	12.5/25	> 100
*Lb. paracasei* L2.2	> 100	25/50	> 100	> 100	**25/25**	> 100	> 100	12.5/25	> 100
*Lb. rhamnosus* L41.2	> 100	> 100	> 100	> 100	**6.25/6.25**	> 100	> 100	25/25	> 100
*En. durans* S1.41	> 100	> 100	6.25/12.5	25/25	**6.25/25**	12.5/50	> 100	12.5/25	> 100
*En. faecalis* S2.43	> 100	> 100	> 100	> 100	**12.5/25**	> 100	> 100	12.5/25	25/25

Values were calculated as the mean of three independent repeats. Results for the ethanolic *O. vulgare* extract (E5), which was selected for further analysis, are marked in bold.

### *In vitro* biocompatibility of the *Origanum vulgare* ethanolic extract

**Figure 2:**
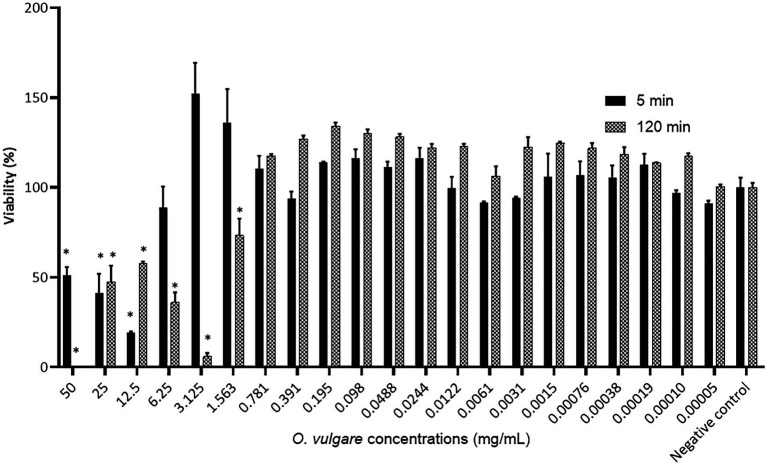
Percentage viability of the human oral keratinocyte cell line B11 after 5 min and 120 min exposure to different concentrations of the *O. vulgare* ethanolic extract. Results were calculated as the means ± SD of three independent repeats. The bars with significant differences from the control were indicated with asterisks. *: (*p* value <0.1).

Since plant-derived biological compounds could also possess toxic activity against eukaryotic cells, the biocompatibility of the *O. vulgare* ethanolic extract was further evaluated by quantifying the cytotoxicity of this extract against human oral keratinocytes. The results are shown in Figure 2 and indicate that a 120 min exposure of the human oral keratinocyte cell line B11 to the *O. vulgare* ethanolic extract (50–1.563 mg/ml) induced a significant reduction in the cell viability compared to the negative control. On the contrary, no reduction in the cell viability was observed at concentrations ≤0.781 mg/ml. With a shorter time of exposure (5 min), the tested cell line was more tolerant to higher concentrations of the *O. vulgare* ethanolic extract; the cell viability was only reduced significantly at concentrations ≥12.5 mg/ml. Based on the results obtained in this section, all the subsequent assays were performed using three biocompatible concentrations (0.781, 0.391 and 0.195 mg/ml).

### In-depth characterization of the anti-biofilm potential of the *Origanum vulgare* ethanolic extract

The MIC values (1.563–25 mg/ml; Table 3) of the *O. vulgare* ethanolic extract against the dental plaque isolates suggested that the extract was unable to completely inhibit planktonic growth at biocompatible concentrations ≤0.781 mg/ml. Nonetheless, it remained possible that the extract had a significant impact on planktonic growth in this concentration range that was not captured by the MIC assays, which only considered complete growth inhibition. To further quantify the antimicrobial activity of biocompatible extract concentrations, the planktonic growth of eight oral isolates, which were specifically selected for their biofilm forming capacity, was determined after 24 h of incubation in the presence of the extract Supplementary Figure S2 (Supplementary material). Only the highest biocompatible concentration of 0.781 mg/ml showed a significant antimicrobial activity against some of the tested isolates, including *Str. salivarius* SA3, *Str. vestibularis* S2P1, *En. durans* S1.41 and *En. faecalis* S2.43. The lower concentrations of 0.390 mg/ml and 0.195 mg/ml did not significantly affect the growth of the tested isolates, except for the concentration of 0.390 mg/ml against *En. faecalis* S2.43. Despite the limited effect of the biocompatible extract concentrations on planktonic growth, more specific anti-biofilm activities could not be excluded.

Given the importance of microbial biofilms in the context of oral health and the promising anti-biofilm effects of the *O. vulgare* ethanolic extract observed in the initial screening assays, the preventive (3.4.1) and curative (3.4.2) anti-biofilm potential of this extract at biocompatible concentrations was further characterized.

### Ability of the *Origanum vulgare* ethanolic extract to prevent oral biofilm formation

**Figure 3:**
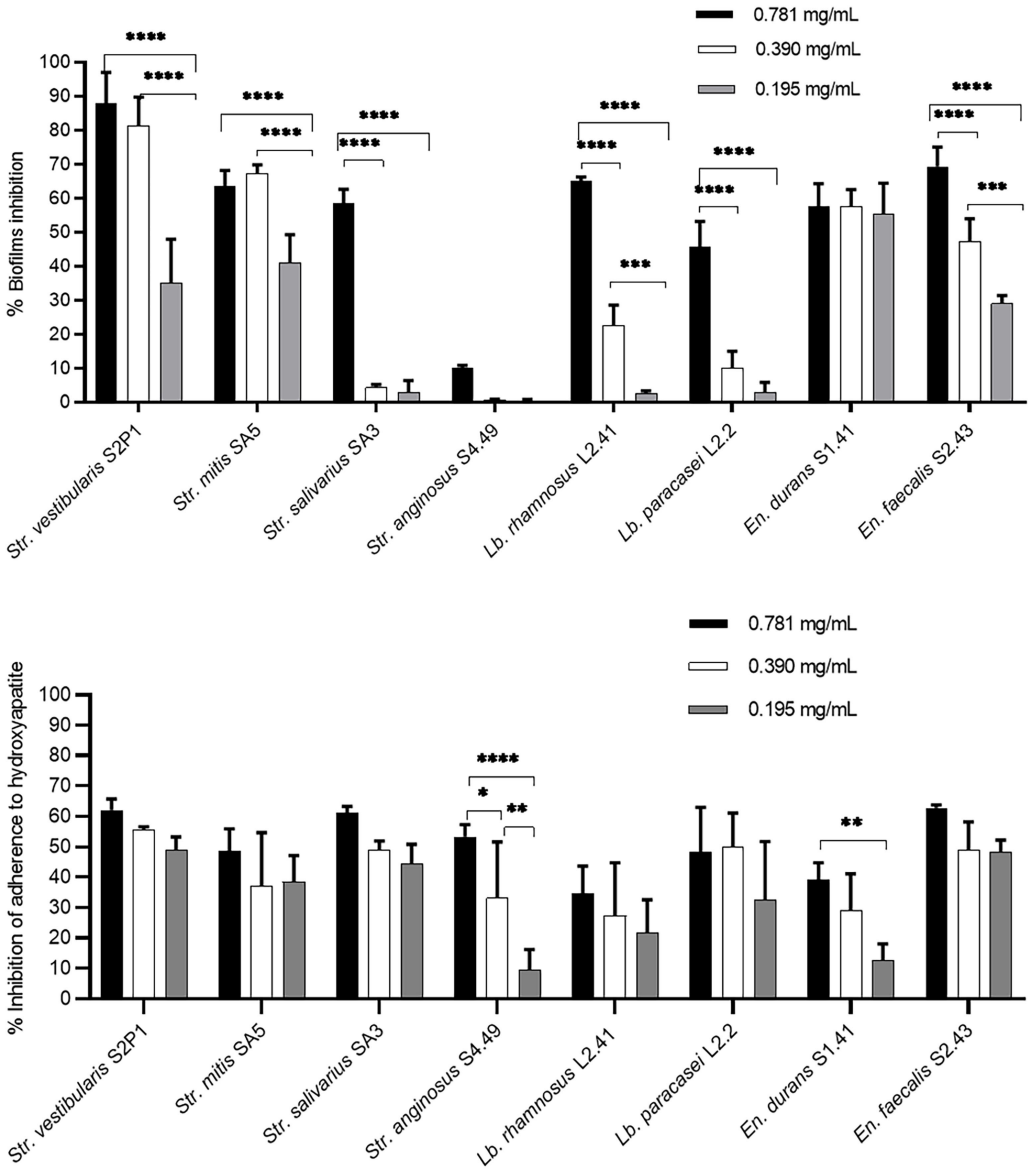
Preventive anti-biofilm potential of the *O. vulgare* ethanolic extract. **(A)** Inhibitory effect of the extract on biofilm formation by the dental plaque isolates on a polystyrene surface. **(B)** Inhibitory effect of the extract on the initial adherence of the dental plaque isolates to hydroxyapatite-coated surfaces. Results were calculated as the means ± SD of three independent repeats. The bars with significant differences were indicated with asterisks. *: (*p* value <0.1), **: (*p* value <0.01), ***: (*p* value <0.001), ****: (*p* value <0.0001).

First, the potential of the *O. vulgare* ethanolic extract to prevent biofilm formation of the eight oral isolates selected for their biofilm forming capacity was evaluated. Bacterial cells were mixed with non-cytotoxic concentrations (0.781 mg/ml, 0.390 mg/ml and 0.195 mg/ml) of the extract and incubated in a polystyrene microtiter plate, after which biofilm formation was quantified using crystal violet and compared to a negative control (Figure 3A). The percentages of biofilm inhibition were found to be high for *Str. vestibularis* S2P1, *Str. mitis* SA5 and *En. faecalis* S2.43, with significant differences between the three concentrations. Similarly, high percentages of biofilm inhibition were observed with *En. durans* S1.41, but no significant difference (*p* > 0.1) was observed between the three extract concentrations. In contrast, high percentages of biofilm inhibition were only observed at the concentrations of 0.781 mg/ml and 0.390 mg/ml in case of *Lb. rhamnosus* L41.2 and at 0.781 mg/ml in case of *Str. salivarius* SA3 and *Lb. paracasei* L2.2. Only for *Str. anginosus* S4.49, no significant inhibition in biofilm formation was observed.

Given the observed *in vitro* anti-biofilm effect, the ability of the *O. vulgare* ethanolic extract to prevent oral biofilm development in a model that more closely resembled the oral niche was validated. Hereto, the ability of the extract to interfere with the adherence of the eight oral isolates to a hydroxyapatite-coated surface, which mimics human enamel, was evaluated (Figure 3B). Also in this model, the three tested *O. vulgare* extract concentrations (0.781, 0.390 and 0.195 mg/ml) showed remarkable anti-biofilm potential and significantly inhibited the adherence to hydroxyapatite for all eight isolates tested. This is also true for *Str. anginosus* S4.49, for which the extract showed only poor biofilm inhibition when grown on a polystyrene surface (Figure 3A). For most isolates, no significant difference (*p* > 0.1) was observed between the three tested concentrations. Only for *Str. anginosus* S4.49 and *En. durans* S1.41, higher extract concentrations performed significantly better compared to lower concentrations.

#### Ability of the *Origanum vulgare* ethanolic extract to eradicate pre-formed oral biofilms

**Figure 4:**
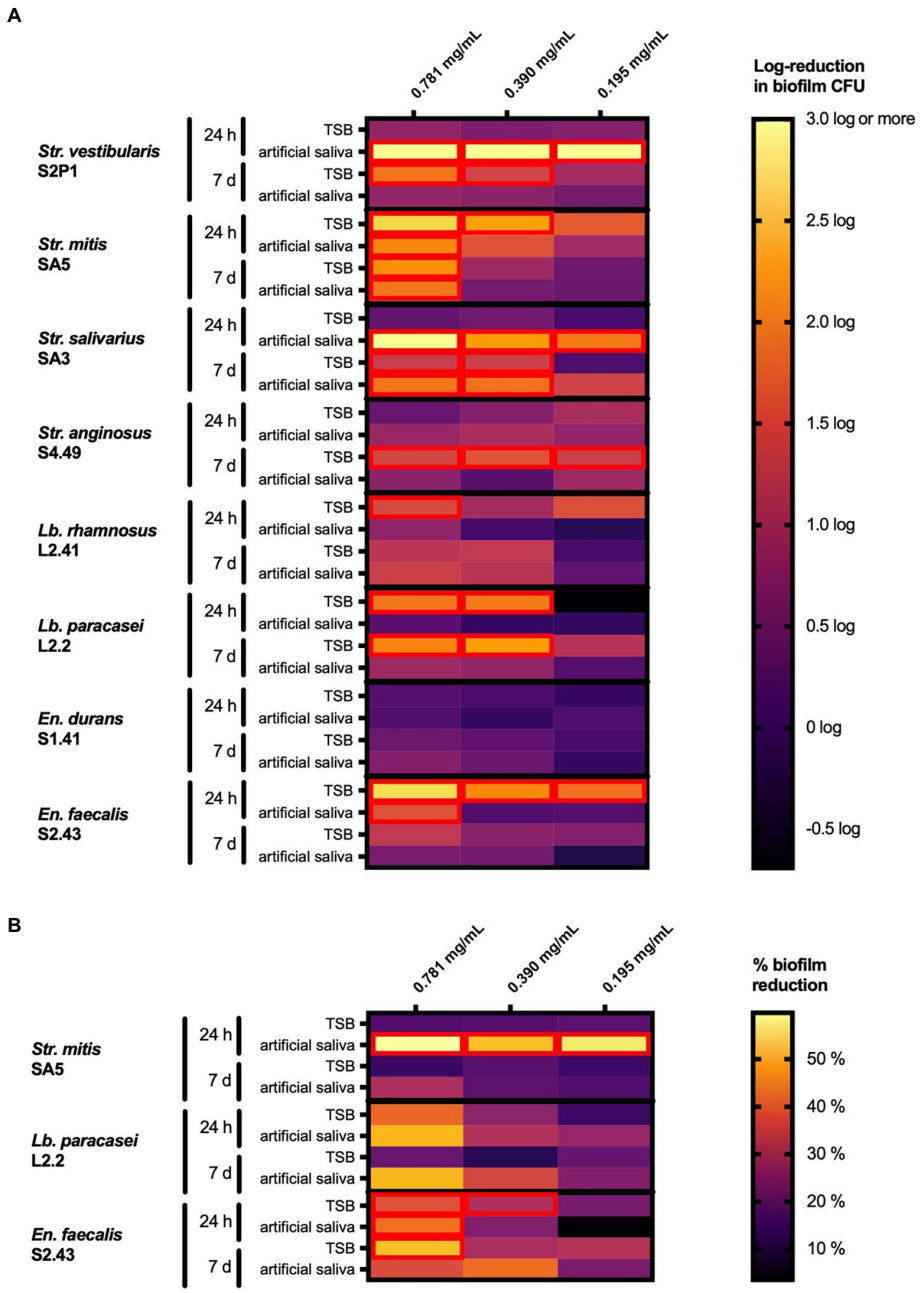
Heat maps representing the effect of the *O. vulgare* ethanolic extract on biofilms of the indicated dental plaque isolates growing on **(A)** polystyrene microplates or **(B)** hydroxyapatite-coated microplates. Biofilms were pre-formed for 24 h or 7 days in either TSB broth or in artificial saliva and were treated for 2 h with the *O. vulgare* extract. In **(A)**, results are represented as a log reduction in biofilm cell counts of treated samples compared to a negative control. In **(B)**, results are represented as a % reduction in bacterial biofilms compared to a negative control as determined with a resazurin-based cell viability assay. Results were calculated as the means of three independent repeats and are presented in more detail in Supplementary Figures S3–S6 (Supplementary material). Cells with a significant reduction (*p* < 0.1) compared to the negative control are marked with a red border.

To demonstrate the curative potential of the extract, its ability to eradicate pre-formed biofilms was further investigated. The data are summarized in Figure 4 and presented in more detail in Supplementary Figures S2–S5 (Supplementary material). Biofilms pre-formed on a polystyrene surface (Figure 4A) for 24 h in TSB were significantly reduced compared to the negative control using the three tested concentrations (0.781, 0.390 and 0.195 mg/ml) for *En. faecalis* S2.43, using two concentrations (0.781 and 0.390 mg/ml) for *Str. mitis* SA5 and *Lb. paracasei* L2.2, and using one concentration (0.781 mg/ml) for *Lb. rhamnosus* L41.2. No significant (*p* > 0.1) biofilm reduction was observed for *Str. salivarius* SA3, *Str. vestibularis* S2P1, *En. durans* S1.41 and *Str. anginosus* S4.49 for these conditions (24-h, TSB). If the biofilms were pre-formed for 24 h in artificial saliva instead of TSB, a significant reduction for all three tested extract concentrations (0.781, 0.390 and 0.195 mg/ml) was observed for *Str. salivarius* SA3 and *Str. vestibularis* S2P1. For *Str. mitis* SA5 and *En. faecalis* S2.43, only an extract concentration of 0.781 mg/ml significantly reduced the bacterial biofilm, while no significant (*p* > 0.1) biofilm reduction was observed for *Str. anginosus* S4.49, *Lb. paracasei* L2.2, *Lb. rhamnosus* L41.2 and *En. durans* S1.41 under these conditions (24-h, artificial saliva).

Noticeably different results were obtained when the period of biofilm formation was extended from 24 h to 7 days. When pre-formed in TSB, these mature 7-day biofilms were significantly reduced compared to the negative control using the three tested extract concentrations (0.781, 0.390 and 0.195 mg/ml) for *Str. anginosus* S4.49. Under these conditions (7-days, TSB), biofilms of *Str. salivarius* SA3, *Str. vestibularis* S2P1 and *Lb. paracasei* S2.2 were reduced after treatment with extract concentrations of 0.781 and 0.390 mg/ml, while only a concentration of 0.781 mg/ml was effective against biofilms formed by *Str. mitis* SA5. No significant (*p* > 0.1) biofilm reduction was observed using any of the three extract concentrations for *Lb. rhamnosus* L41.2, *En. durans* S1.41 and *En. faecalis* S2.43. If artificial saliva was used instead of TSB, only the 7-day biofilms formed by *Str. mitis* SA5 and *Str. salivarius* SA3 were significantly reduced at extract concentrations of 0.781 mg/ml and 0.781 to 0.390 mg/ml, respectively.

In a similar fashion, strikingly different results were obtained when biofilms were pre-formed on a hydroxyapatite-coated surface (Figure 4B). For the 24-h biofilms pre-formed in TSB, only the *En. faecalis* S2.43 biofilm was significantly reduced after treatment with extract concentrations of 0.781 and 0.390 mg/ml. If biofilms were pre-formed for 24 h in artificial saliva, a significant reduction after extract treatment was observed for both *Str. mitis* SA5 (0.781 mg/ml, 0.390 mg/ml and 0.195 mg/ml) and *En. faecalis* S2.43 (0.781 mg/ml). Seven-day biofilms pre-formed on a hydroxyapatite-coated surface in either TSB or artificial saliva were more resistant to the *O. vulgare* ethanolic extract, since only the biofilm of *En. faecalis* S2.43 formed in TSB was significantly reduced when treated with a concentration of 0.781 mg/ml.

### Characterization of the anti-virulence potential of the *Origanum vulgare* ethanolic extract

#### Effect of the *Origanum vulgare* ethanolic extract on glucosyltransferase activity

**Figure 5:**
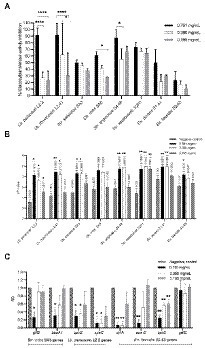
Anti-virulence potential of the *O. vulgare* ethanolic extract. **(A)** Inhibitory effect of the extract on glucosyltransferase activity of dental plaque isolates. **(B)** Inhibitory effect of the extract on organic acid biosynthesis of dental plaque isolates. **(C)** Inhibitory effect of the extract on the expression of virulence-associated genes of *Str. mitis* SA5 (*gtfB*, *bapA1*), *Lb. paracasei* L2.2 (*spxB*, *spaC*) and *En. faecalis* S2.43 (*cylA*, *aceE*, *sprE*, *gelE*). Results were calculated as the means ± SD of three independent repeats. The bars with significant differences were indicated with asterisks. *: (*p* value <0.1), **: (*p* value <0.01), ***: (*p* value <0.001), ****: (*p* value <0.0001).

Glucosyltransferases (Gtfs) are a class of enzymes responsible for the biosynthesis of a variety of glucans. Since glucans are a crucial component of cariogenic biofilms, Gtfs are considered a key virulence factor for caries and other oral infections (Xu et al., 2018). To evaluate the ability of the *O. vulgare* ethanolic extract to inhibit Gtf activity, the biosynthesis of water insoluble glucans was quantified in extract-treated and control samples (Figure 5A). The data confirm that the extract was able to inhibit the Gtf activity for all eight tested isolates. The intensity of inhibition varied between the different isolates and was dependent on the extract concentration used. The inhibition was strongest for all isolates at an extract concentration of 0.781 mg/ml. At this concentration, the highest inhibition percentages were observed for the *Lacticaseibacillus* isolates (91.40–91.66%), followed by the *Streptococcus* isolates (50.00–87.34%). The Gtf activity of the *Enterococcus* isolates was noticeably less influenced by the *O. vulgare* extract, with inhibition percentages between 23.56 and 50.00% for the highest extract concentration tested. At lower extract concentrations, inhibition of water insoluble glucan synthesis was still observed. For an extract concentration of 0.390 mg/ml, the strongest inhibition was observed for *Str. vestibularis* (67.39%) and the lowest for *En. faecalis* (16.66%). Inhibition percentages between 10.91 and 43.47% were obtained with an extract concentration of 0.195 mg/ml.

#### Effect of the *Origanum vulgare* ethanolic extract on biosynthesis of organic acids

In addition to Gtf activity, the ability of oral microorganisms to produce large amounts of organic acids is known to modulate enamel demineralization and initiate dental lesions (Philip and Walsh, 2019). Consequently, the acidification capacity of oral microorganisms can also be considered as a virulence factor in the context of oral health. Therefore, it was evaluated whether the *O. vulgare* ethanolic extract was able to mitigate the ability of oral isolates to acidify culture medium (Figure 5B). In general, the acidification of the culture medium was significantly (*p* < 0.1) reduced upon treatment with 0.781 mg/ml of the extract. Near neutral pH values ranging between 6.16 and 6.96 were recorded for all tested isolates compared to the more acidic pH values (3.56–5.77) obtained in the absence of the plant extract. A reduction in the acidifying activity (pH = 5.63–6.99) was also observed with 0.391 mg/ml of the extract, which was significant (*p* < 0.1) for most but not all isolates. On the contrary, an extract concentration of 0.195 mg/ml induced smaller modifications in the pH values. Only for *Lb. paracasei* L2.2 and *Str. vestibularis* S2P1, a significant increase in pH was observed compared to an untreated control.

#### Effect of the *Origanum vulgare* ethanolic extract on expression of virulence-associated genes

Finally, the effect of the *O. vulgare* ethanolic extract on the expression of virulence-associated genes was investigated. The selected genes include a pyruvate oxidase (*spxB*) and a pilus component (*spaC*) for *Lb. paracasei* L2.2, a glucosyltransferase (*gtfB*) and a biofilm associated protein (*bapA1*) for *Str. mitis* SA5, and a gelatinase (*gelE*), a cytolysin (*cylA*), a collagen adhesin (*aceE*) and a serine protease (*sprE*) for *En. faecalis* S2.43. The results are presented in Figure 5C. Strikingly, the highest extract concentration (0.781 mg/ml) was able to significantly reduce the expression levels of all but one of the eight tested virulence genes. Also the lower extract concentrations, 0.390 and 0.195 mg/ml, significantly reduced the expression levels of four (*spxB*, *spaC*, *cylA*, *sprE*) and one (*sprE*) virulence gene(s) respectively. Only expression of the *En. faecalis* S2.43 *gelE* gene was not significantly affected by any of the three tested extract concentrations.

### Fractionation of the *Origanum vulgare* ethanolic extract and characterization of the pure compounds

**Figure 6:**
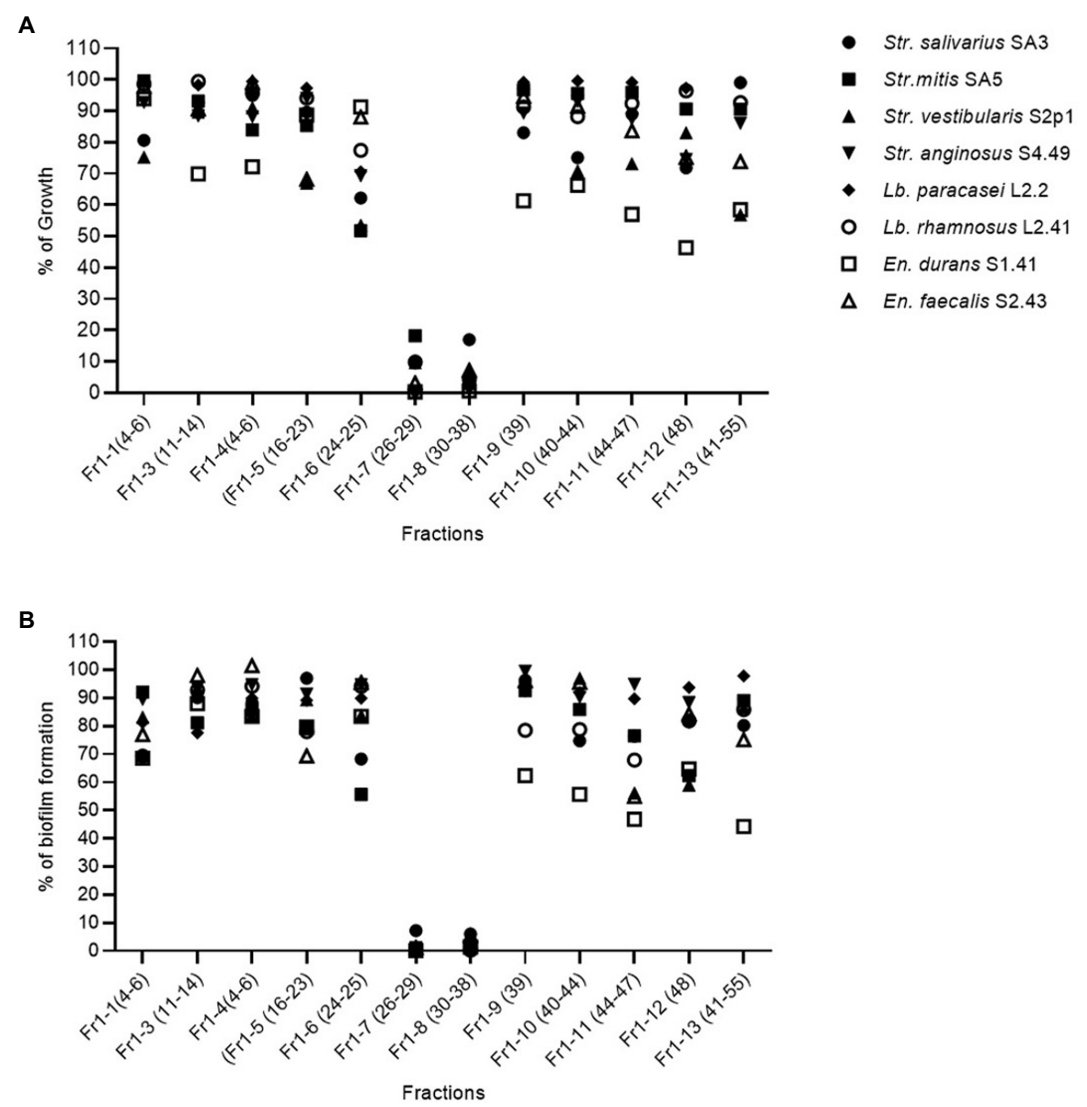
Antibacterial **(A)** and anti-biofilm **(B)** activity of the 12 sub-fractions of the *O. vulgare* ethanolic extract on a panel of eight oral isolates. Results are represented as the % of growth and biofilm formation calculated as the means of three independent repeats. The fractions were obtained by C18 reverse phase column chromatography of the Fr1 fraction.

**Figure 7:**
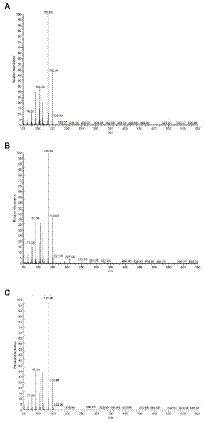
GC–MS chromatograms of Fr1-7 **(A)**, Fr1-8 **(B),** and pure crystalized thymol **(C)**.

Given the interesting antimicrobial, anti-biofilm and anti-virulence properties of the *O. vulgare* ethanolic extract, the active compounds contained within this extract were further investigated. To this end, the extract was fractionated with column chromatography based on the polarity of the components. The antibacterial activity of the four obtained fractions (Fr1 to Fr4) was evaluated against the previously selected panel of eight clinical isolates by the agar disc diffusion method. The results are summarized in Supplementary Table S6 (Supplementary material). Interestingly, all four fractions were active against at least four of the eight tested isolates, indicating that all fractions contained biologically active compounds. Especially fraction Fr1 (eluted with 100% heptane) showed promising antimicrobial properties with inhibition zones ranging from 9 to 20 mm against all eight tested isolates and was selected for additional analysis. This fraction was further purified and 12 sub-fractions were obtained after fractionation with C18 reverse phase column chromatography. The effect of those 12 sub-fractions on growth and biofilm formation of the panel of eight oral pathogens is shown in Figure 6. Compared to the other sub-fractions, the Fr1-7 and Fr1-8 fractions showed strong inhibitory effects on both the planktonic growth and biofilm formation. Nevertheless, weak antibacterial and anti-biofilm activities were also observed with the other fractions against some of the tested isolates, suggesting that there were other active compounds involved in the total effect. To identify the chemical compounds present in the Fr1-7 and Fr1-8 fractions, they were analyzed using GC–MS. The results of the GC–MS analysis presented in Figure 7 indicated that both the Fr1-7 and Fr1-8 chromatograms were highly similar to one another and closely resembled the chromatogram obtained with pure thymol. This suggests that both the Fr1-7 and Fr1-8 fraction mainly contain thymol as their active component.

**Figure 8:**
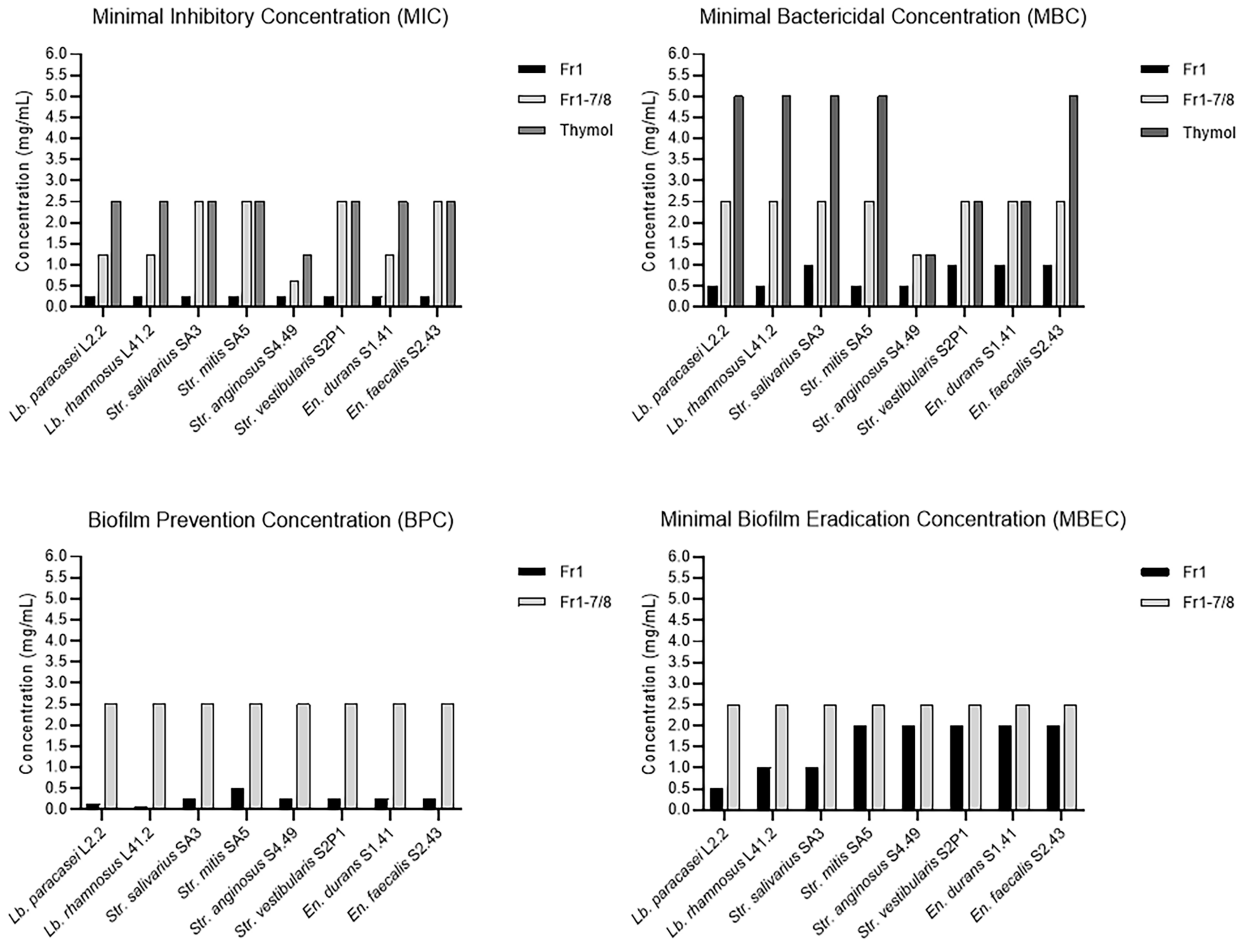
Antibacterial and anti-biofilm activities of the Fr1, Fr1-7, and Fr1-8 fractions of the *O. vulgare* ethanolic extract compared to pure crystalized thymol. The Minimal Inhibitory Concentration (MIC) and Minimal Bactericidal Concentration (MBC) were defined in section 3.2.4. The Biofilm Prevention Concentration (BPC) is defined as the lowest extract concentration at which no biofilm formation occurred when bacterial inoculation and extract addition were performed simultaneously. The Minimal Biofilm Eradication Concentration (MBEC) is defined as the lowest extract concentration that significantly eradicated pre-formed biofilms.

This hypothesis was further investigated by comparing the antibacterial and anti-biofilm activities of the Fr1, Fr1-7 and Fr1-8 fractions to the activities observed with pure crystalized thymol (CAS: 89–83-8) against a panel of eight dental plaque isolates. The results are shown in Figure 8. Comparing the antibacterial and anti-biofilm activities obtained with thymol to those obtained with the Fr1-7/Fr1-8 fractions, the active concentrations were relatively similar. The MIC values ranged between (0.625–2.5 mg/ml) and (1.25–2.5 mg/ml) with Fr1-7/Fr1-8 and thymol, respectively. Similarly, the MBC values were (1.25–2.5 mg/ml) and (1.25–5.0 mg/ml) for the Fr1-7/Fr1-8 fractions and thymol, respectively. The biofilm-prevention concentration (PBC) and minimal biofilm eradication concentration (MBEC) values were both 2.5 mg/ml in case of Fr1-7/Fr1-8, whereas the biofilm bactericidal concentration (BBC), defined as the lowest extract concentration producing a 3 log_10_ reduction in biofilm CFU counts, of thymol was 1.25 mg/ml. Taken together, these results reinforce the hypothesis that thymol is the main active component of the Fr1-7 and Fr1-8 fractions. Nonetheless, for some isolates the antimicrobial activity of the Fr1-7 and Fr1-8 fractions (as quantified by the MIC and MBC values) was slightly higher compared to pure thymol. This could indicate that the Fr1-7 and Fr1-8 fractions were not completely pure and were still contaminated with small amounts of other bioactive compounds. Interestingly, when comparing the results for the Fr1-7 and Fr1-8 fractions with those obtained with Fr1, the latter consistently showed a remarkably stronger antimicrobial and anti-biofilm effect across the different clinical isolates (Figure 8). This observation suggests that in addition to sub-fractions Fr1-7 and Fr1-8, also other sub-fractions of Fr1 contain bioactive compounds. Furthermore, since these other sub-fractions only showed weak antimicrobial and anti-biofilm activity by themselves (Figure 6), this implicates a synergistic activity between thymol and one or more unidentified compounds contained within the Fr1 fraction.

## Discussion

Dental caries and subsequent oral infections remain a challenge for oral health, especially given the lack of effective and safe treatment options that are currently available (Palombo, 2011; Moghadam et al., 2020). In the established dental caries paradigm, progression of caries is subdivided into three stages. First, oral streptococci (in particular mutans streptococci) colonize the tooth surface and create a low pH environment caused by carbohydrate fermentation. This low pH niche creates a selective environment that favors colonization of other acid-tolerant and acid-producing bacteria including lactobacilli. This in turn, further increases acid formation, eventually resulting in enamel demineralization and the onset of dental lesions (Caufield et al., 2015). In agreement with this model, the majority of the 83 dental plaque isolates from Algerian patients obtained in this study were either streptococci or lactobacilli. While the microbial composition of dental plaques of Algerian patients has been described before, the most recent report is over 5 years old (Hoceini et al., 2016). More up-to-date data is therefore welcome to guide oral health research towards bacterial species that are currently the most relevant problem for the Algerian population. Indeed, none of the 10 *Streptococcus* isolates that were identified with 16S rDNA sequencing (*Str. mitis*, *Str. salivarius*, *Str. anginosus* and *Str. vestibularis*) belonged to the mutans *Streptococcus* group, which is in contrast with previous results on Algerian dental plaque composition (Hoceini et al., 2016). A low abundance of mutans streptococci in dental lesions has been previously reported (Simón-Soro et al., 2014) and questions the importance of this group in the current dental caries paradigm. Among the 20 lactobacilli isolated in this study, only two species were identified: *Lb. paracasei* and *Lb. rhamnosus*. It was already reported that species of the *Lb. casei* group are predominant in oral biofilms and tooth enamel lesions of children with early childhood caries (Neves et al., 2017; Reis et al., 2021). In addition, *Lb. paracasei* was found as the most prevalent species of lactobacilli in carious dentin followed by *Lb. rhamnosus* (Damé-Teixeira et al., 2021). In addition to streptococci and lactobacilli, *En. faecalis* and *En. durans* were isolated in this study. While the role of enterococci in dental caries is less well understood, they have been previously reported to play a role in oral infections and to form biofilms in root canals (Komiyama et al., 2016).

Alike other countries, medicinal plants have been traditionally used for treating dental pain and oral infections in Algeria. They are considered as an important source of bioactive substances which could be used as safe, effective and economical compounds to control oral biofilms (Palombo, 2011). As the ongoing antimicrobial resistance crisis has underpinned the importance of expanding our antimicrobial drug portfolio, this study aimed to screen 27 extracts of nine Algerian medicinal plants for their antimicrobial and anti-biofilm effects. Overall, the results revealed promising antimicrobial and anti-biofilm properties among the different extracts, with interesting differences depending on (i) the medicinal plant species, (ii) the extraction method and (iii) the target microorganisms. While prior studies have already reported antimicrobial activities against oral pathogens for the selected medicinal plants (*Artemisia herba alba, Centaurium erythraea, Juglans regia, Laurus nobilis, Matricaria recutita, Mentha pulegium, Mentha piperita, Origanum vulgare* and *Taraxacum officinale*), the screening approach in the current study further elaborates on two important elements, being the comparison between different extraction methods and the use of an extensive library of dental plaque isolates (Marzouki et al., 2009; Pereira et al., 2011; Chaiya et al., 2013; Zakavi et al., 2013; Krumina et al., 2015; Sangeetha and Ezhilarasan, 2016; Arbia et al., 2017; Hickl et al., 2018; Tardugno et al., 2018).

First, our screening provides a systematic comparison between the antimicrobial and anti-biofilm activities of ethanolic versus aqueous extracts. For most assayed plants, ethanolic extracts provided a stronger activity in the initial screening assays, which was further confirmed by the MIC/MBC assay as all aqueous extracts had MIC/MBC values >100 mg/ml against all tested dental plaque isolates. A similar observation was also reported by Barbour et al. (2004) after screening an extensive collection of 27 Lebanese medicinal plants for antimicrobial activity against nine common pathogens. Taken together, these observations suggest that organic solvents are more suitable to extract compounds with antimicrobial potential from medicinal plants. Nevertheless, it remains possible that aqueous extracts outperform organic extracts for some plant/pathogen combinations. This is illustrated by the aqueous infusion extract of *M. recutita* (I2), which showed stronger antimicrobial and anti-biofilm activities compared to the ethanolic extract (E2) for the majority of oral isolates. This observation underscores the importance of including multiple extraction strategies when attempting to isolate bioactive compounds from medicinal plants.

Second, our screening has included an extensive library of clinically relevant and strong biofilm producing dental plaque isolates. In contrast, most research investigating the selected plants in the context of oral health has used a limited panel of laboratory reference strains to demonstrate antimicrobial properties (Albuquerque et al., 2010; Pereira et al., 2011; Chaiya et al., 2013; Zakavi et al., 2013; Krumina et al., 2015; Vaca et al., 2021). The advantage of including a broad range of clinical isolates is that it gives important knowledge on the activity spectrum of the plant extracts. For example, the ethanolic (E5) and aqueous maceration (M5) extracts of *O. vulgare* were active against all included isolates. Similarly, the ethanolic extract of *J. regia* (E8) showed broad spectrum activity against almost all included isolates, while the aqueous infusion extract (I8) of this plant showed activity, at least in the agar disc diffusion assay, against all *Streptococcus* and *Enterococcus* isolates, but not against the lactobacilli. Furthermore, Figure 1 demonstrates that the *Str. mutans* ATCC 25175 reference strain is not necessarily a good indicator to predict antimicrobial activity of the plant extracts against oral *Streptococcus* isolates. For example, some extracts (including M4, M5 and I5) showed promising antimicrobial properties against the oral *Streptococcus* isolates while showing poor antimicrobial activity against the *Str. mutans* reference strain. As a result, the antimicrobial potential of these extracts would have been severely underestimated if only the reference strain would have been included in the screening. This highlights the importance of including an extensive library of clinical isolates when characterizing the antimicrobial potential of plant extracts.

Interestingly, specific antimicrobial or anti-biofilm properties have been identified in some combinations of target strain/plant extract (Figure 1). Some extracts exerted strong antimicrobial activity (≥ 80% of growth inhibition) but low anti-biofilm potential. This was observed for example with the ethanolic extracts of *T. officinalis* (E3) (against *Str. salivarius* SA3), *L. nobilis* (E9) (in case of *Str. vestibularis* S2P1), *S. officinalis* (E4) against *C. albicans* ATCC 28366 and *M. piperita* (E7) (against *C. albicans* ATCC 28366). In contrary, some extracts exert higher anti-biofilm potential (≥80% of biofilm inhibition) comparing to their limited antimicrobial activity. For example this was noticed with the aqueous extract of *M. recutita* (M2) (against *Str. salivarius* SA3), the *O. vulgare* ethanolic extract (E5) (against the *Str. mitis* SA5 and the *Lb. paracasei* L2.2), the aqueous extracts of *C. erythraea* (M1), *M. recutita* (M2) and *J. regia* (M8) (against *Str. mutans* ATCC 25175) and the ethanolic extracts of *T. officinalis* (E3) and *J. regia* (E8) (against *C. albicans* ATCC 28366). Such specific anti-biofilm properties are interesting, as we have previously demonstrated that, in contrast to classical antibiotics, biofilm inhibition can be more evolutionarily robust to the development of resistance (Dieltjens et al., 2020).

The ethanolic extracts of *O. vulgare* (E5) and *J. regia* (E8) emerged from our screening as the most promising extracts to combat oral infections with the strongest antimicrobial and anti-biofilm activities against a broad range of dental plaque isolates. The antimicrobial properties of both *O. vulgare* and *J. regia* in the context of oral health have already been investigated. For example, Hickl et al. (2018) have reported MIC and MBC values of 0.6 to 5.0 mg/ml and 1.25 to 5.0 mg/ml, respectively, for a methanolic extract of *O. vulgare* against a reference panel of oral pathogens. Also ethanolic and aqueous extracts of *O. vulgare* have been demonstrated to possess antimicrobial activity against oral streptococci (Schovelin and Muñoz, 2018; Vaca et al., 2021), enterococci (Benbelaïd et al., 2014), lactobacilli (Miller et al., 2014) and *Candida* species (Bhat et al., 2018). In addition to oral pathogens, there has been a large body of work in Algeria on the antimicrobial activity of *O. vulgare* against other relevant pathogens, including *Listeria monocytogenes* (Hazzit et al., 2006), *Escherichia coli* (Nabti et al., 2020; Benameur et al., 2022; Bouaouina et al., 2022), *Klebsiella pneumoniae* (Benameur et al., 2022; Bouaouina et al., 2022), *Acinetobacter baumannii* (Bouaouina et al., 2022), *Staphylococcus aureus* (Heni et al., 2020; Bouaouina et al., 2022) and *Salmonella* spp. (Benameur et al., 2022). Similarly, for *J. regia* both aqueous and ethanolic extracts have shown antibacterial activity against different oral *Streptococcus* (Zakavi et al., 2013), *Enterococcus* (Didi et al., 2019) and *Candida* (Martins et al., 2015) strains. However, to the best of our knowledge, this is the first study to report antimicrobial and anti-biofilm activity of *J. regia* bark extracts against oral lactobacilli and *F. nucleatum*.

As the *O. vulgare* ethanolic extract (E5) consistently showed lower MIC and/or MBC values compared to the *J. regia* ethanolic extract (E8) (Table 3), the former was selected for further characterization. Because plant-derived antimicrobials may exhibit toxic effects in humans, such as genotoxicity, neurotoxicity, and hepatotoxicity (Qiu et al., 2020), the effects of the selected *O. vulgare* ethanolic extract on human oral keratinocytes were evaluated (Figure 2). No reduction in the cell viability was observed at concentrations ≤0.781 mg/ml, confirming the biocompatibility of the extract. These results are in agreement with the work of Savini et al. (2009), who showed that a 24-h treatment of human dermal fibroblasts and colonocytes with 0.300 mg/ml of an *O. vulgare* ethanolic extract did not affect cell viability. Nonetheless, this is the first study reporting on the effect of an ethanolic *O. vulgare* extract on human oral cells. As such, these results provide an important non-cytotoxic concentration range, which is crucial knowledge for the development of safe *O. vulgare*-based healthcare products for oral application.

Given that the initial screening assays were performed with a concentration (12.5 mg/ml) well above the cytotoxicity threshold of 0.781 mg/ml, the antimicrobial properties of the *O. vulgare* ethanolic extract at non-cytotoxic concentrations were further validated. Since the effect of the extract on planktonic growth of the dental plaque isolates were found to be only limited in this concentration range, we focussed on the anti-biofilm potential instead. Previous studies have already demonstrated the anti-biofilm potential of *O. vulgare* extracts against oral bacteria in standard *in vitro* setups (Khan et al., 2017; de Oliveira Carvalho et al., 2020; Badekova et al., 2021). For example, Vaca et al. (2021) showed that biofilms formed by a mixed culture of *Str. mutans* and *Str. sobrinus* were reduced by 91% after treatment with a hydrogel loaded with 2% of an *O. vulgare* ethanolic extract. Similarly, a methanolic *O. vulgare* extract has been reported to reduce biofilm formation of a clinical *Str. mutans* isolate, but only at concentrations above 5 mg/ml (Hickl et al., 2018). Here, this available knowledge is extended by further validating the anti-biofilm effect of *O. vulgare* against a panel of clinically relevant dental plaque isolates under conditions mimicking the oral niche.

It was found that the *O. vulgare* ethanolic extract possessed a broad-spectrum capacity to prevent the development of oral biofilms (Figure 3). Comparing with the results of the antimicrobial activity, in which significant reduction in bacterial growth was only observed at the highest non-cytotoxic concentration of 0.781 mg/ml (against *Str. salivarius* SA3, *Str. vestibularis* S2P1, *En. durans* S1.41 and *En. faecalis* S2.43) and at 0.390 mg/ml (against the *En. faecalis* S2.43) (Supplementary Figure S2, Supplementary material), specific anti-biofilm effects could be suspected. For example, at the two lower concentrations of 0.390 mg/ml and 0.195 mg/ml, no antimicrobial effect was observed against the clinical isolates *Str. vestibularis* S2P1, *En. durans* S1.41 and *En. faecalis* S2.43, while a significant anti-biofilm effect was observed against these isolates. Similarly, while no antimicrobial activity was observed against the two *Lacticaseibacillus* isolates, the *O. vulgare* ethanolic extract was able to prevent biofilm formation of both of *Lb. rhamnosus* L41.2 (0.781 mg/ml and 0.390 mg/ml) and *Lb. paracasei* L2.2 (0.781 mg/ml). Remarkably, no antimicrobial activity was observed against *Str. mitis* SA5, but all three selected extract concentrations were able to reduce biofilm formation. On the contrary, no antimicrobial and anti-biofilm effects were observed against *Str. anginosus* SA5.

Both biofilm formation on polystyrene and initial adherence to hydroxyapatite, which mimics human enamel, were significantly reduced for seven out of eight tested dental plaque isolates. Only biofilm formation on polystyrene by *Str. anginosus* S 4.49 was not affected by extract treatment. Nonetheless, extract treatment reduced the adherence of this isolate to hydroxyapatite by over 50%. This illustrates that simple *in vitro* models might not always accurately reflect the anti-biofilm potential of bioactive compounds in more complex environments like the oral niche. This point is further strengthened by our data on the ability of the extract to eradicate pre-formed biofilms (Figure 4). While the *O. vulgare* ethanolic extract demonstrated the ability to partially eradicate pre-formed biofilms produced by most dental plaque isolates, the observed efficacy was strongly dependent on the parameters of the setup, including the substratum on which the biofilm was formed (polystyrene vs. hydroxyapatite), the growth medium (TSB vs. artificial saliva), and the maturity of the biofilm (24-h vs. 7-days). Taken together, our anti-biofilm data confirms that the *O. vulgare* ethanolic extract shows promise to be developed into a broad-spectrum product to combat oral biofilms. Nonetheless, given the substantial variation in anti-biofilm efficacy that was observed between different setups, this should be further validated *in vivo*.

In addition to these anti-biofilm activities, the anti-virulence properties of the *O. vulgare* ethanolic extract was also investigated by quantifying the expression levels of a set of virulence-associated genes after extract treatment. Moreover, the effects of extract treatment on glucosyltransferase (Gtf) activity and acidogenesis were investigated. Gtfs are extracellular enzymes that in the presence of sucrose synthesize glucans which contribute to biofilm formation. A glucan-rich biofilm matrix creates a rigid barrier that protects the plaque microflora from external antimicrobial substances and provides a source of easily metabolizable carbohydrates. Metabolization of these carbohydrates results in the production of large amounts of organic acids, thus increasing the acidity of the dental plaque biofilms and modulating enamel demineralization (Philip and Walsh, 2019; Harper et al., 2021).

In general, broad-spectrum anti-virulence properties of the *O. vulgare* ethanolic extract were observed against *Lacticaseibacillus*, *Streptococcus* and *Enterococcus* isolates, even at non-cytotoxic concentrations (Figure 5). Treatment with the highest extract concentration (0.781 mg/ml) reduced Gtf activity for ≥50% for all dental plaque isolates tested, except for *En. faecalis* S2.43. In addition, the same extract dose also markedly reduced the expression levels of glucosyltransferase B (*gtfB*) for *Str. mitis* SA5. Together, this curtailment of glucan synthesis following extract treatment could provide a molecular mechanism explaining the observed reduction in biofilm formation of the dental plaque isolates. Nonetheless, the *Streptococcus* isolate that suffered the strongest inhibition of Gtf activity (*Str. anginosus* S4.49) showed the weakest inhibition of biofilm formation. This suggests that inhibition of glucan synthesis by itself is not sufficient to explain the observed anti-biofilm effects. Other biofilm-associated virulence genes, including the *spaC* pilus for the lactobacilli (Reis et al., 2021), a biofilm-associated protein (*bapA1*) for the streptococci (Khan et al., 2016) and a collagen adhesin (*aceE*) for the enterococci (Lee and Tan, 2015), were also significantly downregulated after extract treatment. These effects could further explain the observed reduction in biofilm production.

In addition to the anti-virulence effects targeting biofilm formation, an inhibition of other virulence mechanisms was also observed. Exposure to 0.781 mg/ml of the *O. vulgare* ethanolic extract reduced the acidogenic capacity of the dental plaque isolates, keeping the culture medium at a close-to-neutral pH. Extract treatment also reduced the expression levels of other virulence-associated genes, including a pyruvate oxidase (*spxB*) responsible for H_2_O_2_ production in lactobacilli, a cytolysin (*cylA*) which contributes to the persistence of enterococcal infections by lysing immune cells and a serine protease (*sprE*), which has been shown to facilitate enterococcal adhesion to dentin (Lee and Tan, 2015; Reis et al., 2021). The only virulence-associated gene under study that was not significantly affected by extract treatment was an enterococcal gelatinase (*gelE*), which has been implicated in the degradation of dentin (Lee and Tan, 2015). Although some studies have reported that other bioactive compounds can influence Gtf activity, acidogenesis and expression of the selected virulence-associated genes in cariogenic bacteria, this is the first report that describes those effects for an *O. vulgare* extract (Furiga et al., 2008; Lee and Tan, 2015; Khan et al., 2016; Shafiei et al., 2020; Priya et al., 2021; Reis et al., 2021). Especially against the backdrop of an antimicrobial resistance crisis, anti-virulence properties are believed to be more robust to the development of resistance and provide an attractive complementation to the more conventionally studied bacteriostatic and bactericidal activities (Dickey et al., 2017).

Fractionation and subsequent GC–MS analysis of the *O. vulgare* ethanolic extract has put forward thymol as an important active compound. Thymol is a monoterpene phenol which is well known for its benefits on health. It has demonstrated a plethora of biological activities, including antioxidant, anti-inflammatory, anti-epileptogenic and anti-cancer properties (Sancheti et al., 2014; Islam et al., 2019). Also in the context of oral health, thymol has been extensively studied for its antimicrobial, anti-biofilm and anti-virulence properties against oral pathogens. Because of these interesting properties, thymol has already been proposed as an active compound in numerous products for oral hygiene, including mouthwashes (Mouchrek Junior et al., 2015), varnishes (Jentsch et al., 2014) and dentifrices (Yu et al., 2000). In a recent publication, Priya et al. (2021) show that thymol completely inhibits the growth of a reference strain of *C. albicans* and *Str. mutans* at concentrations of 0.128 mg/ml and 0.256 mg/ml, respectively. Interestingly, these MIC values are substantially lower compared to the MIC values for thymol we obtained against the dental plaque isolates (Figure 8), which ranged from 1.25 to 2.50 mg/ml. This discrepancy further highlights the importance of including clinical isolates when assessing antimicrobial properties of bioactive compounds. In the same study, sub-MIC concentrations of thymol were also shown to prevent biofilm development and adherence to polystyrene surfaces of both the *C. albicans* and *Str. mutans* reference strain. Moreover, thymol showed a dose-dependent reduction in acidogenesis of *Str. mutans* and reduced the expression of multiple virulence-associated genes, including the *gtfB* gene also investigated in this study (Priya et al., 2021). Because these results are reminiscent of the data presented here, this supports our hypothesis that thymol is one of the main active components of the *O. vulgare* ethanolic extract.

Importantly, the Fr1 fraction of the *O. vulgare* ethanolic extract showed stronger antimicrobial and anti-biofilm activities across the panel of clinical isolates compared to both the Fr1-7/Fr1-8 sub-fractions and pure thymol (Figure 8). Since the other sub-fractions of Fr1 only showed weak activity by themselves (Figure 6), this observation suggests that the Fr1 fraction contains other bioactive compounds that act synergistically with thymol. While the chemical composition of the other Fr1 sub-fractions was not further analyzed, others have reported carvacrol, thymol, *p*-cymene, 1-octacosanol, 𝛾-terpinene, rosmarinic acid, quercetin and apigenin as the most bioactive constituents of *O. vulgare* extracts (Kokkini et al., 1997; Coccimiglio et al., 2016; Khan et al., 2017; Chuang et al., 2018; Vaca et al., 2021). The antimicrobial and anti-biofilm potential of carvacrol, thymol, *p*-cymene and 𝛾-terpinene have been already reported against oral pathogenic strains of *Staphylococcus aureus*, *Str. mutans*, *Str. constelatus*, *En. faecium* and *En. faecalis* (Miladi et al., 2017). In addition, carvacrol, thymol, *p*-cymene and 𝛾-terpinene have been previously shown to inhibit efflux pumps in *C. albicans* and/or *S. aureus*, synergistically enhancing the treatment with other antimicrobials (Miladi et al., 2017; Bae and Rhee, 2019). Moreover, synergistic activity between thymol and carvacrol has been previously reported against *En. faecalis* (Gutiérrez-Fernández et al., 2013). Consequently, these other *O. vulgare* active compounds are interesting candidates to explain the observed synergistic effect with thymol. Further research into the exact identity of these bioactive compounds and potential applications of *O. vulgare* ethanolic extracts is recommended.

In conclusion, we report on the systematic screening of 27 medicinal plant extracts against a library of dental plaque isolates. Broad-spectrum antimicrobial and anti-biofilm properties were observed, especially among ethanolic extracts, which marks them as a promising source for bioactive compounds to control oral biofilms. Moreover, the results show differential activity between clinical isolates and reference strains, highlighting the importance of including an extensive collection of clinically relevant isolates when screening plant extracts for antimicrobial activity. The ethanolic extract of *O. vulgare*, which showed the most promising effects in the initial screening, was further characterized and was found to possess broad-spectrum anti-biofilm and anti-virulence properties against dental plaque isolates, even at non-cytotoxic concentrations. While thymol, a phenol which has already been extensively evaluated in the context of oral health, was identified as an important active compound of the extract, other unidentified compounds contributed substantially and synergistically to the activity of the extract. Further research into the bioactive compounds and potential applications of the *O. vulgare* ethanolic extract could yield novel products to fight dental caries and periodontal diseases.

## Data availability statement

The 16S rDNA sequences of the clinical isolates have been deposited in the GenBank repository with accession numbers ON478995-ON479034.

## Author contributions

FI performed all experimental work and the subsequent data analysis, except for the fractionation, and purification of the *O. vulgare* ethanolic extract (which was performed by GC) and the biocompatibility experiment (which was performed by DG). FI wrote the first draft of the manuscript, which was later revised by SG, DG, FB, and HS. HS and FB supervised the project. All authors contributed to the interpretation of the results and provided valuable feedback on the manuscript. All authors contributed to the article and approved the submitted version.

## Funding

FI received a PhD scholar fellowship from the Algerian-government through the PNE (Programme National Exceptionnel) program. We thank the “Direction Génerale de la Recherche Scientifique et du Developpement Technologique” (DGRSDT) of the High Education and Scientific Research Ministry for supporting our research. This work was further supported by the KU Leuven Research Fund (C24/18/046, C32/17/020 and C3/20/081), and by Research Foundation Flanders (FWO) under grant 3G046318 and grant FWO-SBO S007019N (Bisceps).

## Conflict of interest

The authors declare that the research was conducted in the absence of any commercial or financial relationship that could be construed as a potential conflict of interest.

## Publisher’s note

All claims expressed in this article are solely those of the authors and do not necessarily represent those of their affiliated organizations, or those of the publisher, the editors and the reviewers. Any product that may be evaluated in this article, or claim that may be made by its manufacturer, is not guaranteed or endorsed by the publisher.

## Footnotes

^1^ http://blast.ncbi.nlm.nih.gov
