# Supplementary material for: Origanum vulgare ethanolic extracts as a promising source of compounds with antimicrobial, anti-biofilm, and anti-virulence activity against dental plaque bacteria
Source: Front Microbiol. 2022 Nov 2;13:999839. doi: 10.3389/fmicb.2022.999839 (PMC9668103; doi:10.3389/fmicb.2022.999839)
Supplement: Supplementary file 1 [file Data_Sheet_1.docx]

Supplementary Material

# Results

## Screening of the bacterial isolates for their biofilm formation potential and their molecular identification

|  |
| --- |
|  |

**Figure S1.** Biofilm formation capacity of the most relevant bacterial isolates as determined by crystal violet staining. Results were calculated as the means ± SD of the three independent repeats and were analyzed using one-way ANOVA. The bars with significant differences from the control were indicated with asterisks. *: (*P* value < 0.1), **: (*P* value < 0.01). ***: (*P* value < 0.001). ****: (*P* value < 0.0001).

**Table S1.** Identification of the 40 selected bacterial isolates using 16S rDNA sequencing.

| **isolate code** | **16S rDNA fragment length** | **Identification** | **% of identity** | | **isolate code** | **16S rDNA fragment length** | **Identification** | **% of identity** | |
| --- | --- | --- | --- | --- | --- | --- | --- | --- | --- |
| **L14** | 878 | *Lacticaseibacillus rhamnosus* | | 99.9 | **SA3** | 955 | *Streptococcus salivarius* | | 99.8 |
| **L15** | 897 | *Lacticaseibacillus rhamnosus* | | 99.9 | **S2.44** | 1420 | *Streptococcus mitis* | | 99.7 |
| **L16** | 1459 | *Lacticaseibacillus rhamnosus* | | 100 | **S1.43** | 892 | *Streptococcus mitis* | | 100 |
| **L21** | 1401 | *Lacticaseibacillus rhamnosus* | | 100 | **SA5** | 918 | *Streptococcus mitis* | | 100 |
| **L27** | 847 | *Lacticaseibacillus rhamnosus* | | 100 | **S4.49** | 1012 | *Streptococcus anginosus* | | 99.5 |
| **L2.2** | 826 | *Lacticaseibacillus paracasei* | | 99.9 | **S4.1** | 997 | *Streptococcus anginosus* | | 99.3 |
| **L2** | 1453 | *Lacticaseibacillus rhamnosus* | | 99.9 | **S1.22** | 986 | *Streptococcus mitis* | | 99.8 |
| **L33** | 1456 | *Lacticaseibacillus rhamnosus* | | 100 | **SA2** | 869 | *Streptococcus mitis* | | 100 |
| **L36** | 1462 | *Lacticaseibacillus rhamnosus* | | 99.9 | **S1.49** | 976 | *Streptococcus mitis* | | 99.9 |
| **L41.1** | 904 | *Lacticaseibacillus rhamnosus* | | 100 | **S2P1** | 879 | *Streptococcus vestibularis* | | 99.3 |
| **L41.2** | 882 | *Lacticaseibacillus rhamnosus* | | 100 | **S1.41** | 888 | *Enterococcus durans* | | 99.9 |
| **L46** | 1460 | *Lacticaseibacillus rhamnosus* | | 100 | **S2P2** | 824 | *Enterococcus faecalis* | | 99.9 |
| **L50** | 1344 | *Lacticaseibacillus rhamnosus* | | 99.9 | **S3P2** | 891 | *Enterococcus faecalis* | | 99.8 |
| **L51** | 1460 | *Lacticaseibacillus rhamnosus* | | 100 | **S2.43** | 1337 | *Enterococcus faecalis* | | 99.6 |
| **L6** | 1400 | *Lacticaseibacillus paracasei* | | 100 | **S4.46** | 1450 | *Enterococcus faecalis* | | 99.9 |
| **L55** | 956 | *Lacticaseibacillus rhamnosus* | | 99.7 | **S5.49** | 1454 | *Enterococcus faecalis* | | 99.9 |
| **L17** | 826 | *Lacticaseibacillus rhamnosus* | | 97.8 | **SF1** | 1452 | *Enterococcus faecalis* | | 99.8 |
| **L2.22** | 826 | *Lacticaseibacillus paracasei* | | 98.7 | **S1.33** | 949 | *Enterococcus faecalis* | | 99.8 |
| **L2.26** | 824 | *Lacticaseibacillus paracasei* | | 99.9 | **S1.72b** | 896 | *Enterococcus faecalis* | | 99.8 |
| **L11** | 826 | *Lacticaseibacillus rhamnosus* | | 100 | **S1.63b** | 1451 | *Enterococcus faecalis* | | 99.9 |

## Screening of plant extracts for their antimicrobial and anti-biofilm activities

### Screening the antimicrobial activity of the plant extracts following an agar disc diffusion approach

**Table S2**: Antibacterial activity of the plant extracts on the *Streptococcus* and *Enterococcus* isolates as determined with the agar disc diffusion assay. The diameters of the inhibition zones (mm) were calculated as the mean of three independent repeats. E: ethanolic extract; I: aqueous extract obtained by infusion; M: aqueous extract obtained by maceration.

|  | ***Str. salivarius* SA3** | ***Str. mitis* S2.44** | ***Str. mitis* S1.43** | ***Str. mitis* SA5** | ***Str. anginosus* S4.49** | ***Str. anginosus* S4.1** | ***Str. mitis* S1.22** | ***Str. mitis* SA2** | ***Str. mitis* S1.49** | ***Str. vestibularis* S2P1** | ***En. durans* S1.41** | ***En. faecalis* S2P2** | ***En. faecalis* S3P2** | ***En. faecali*s S2.43** | ***En. faecalis* S4.46** | ***En. faecalis* S5.49** | ***En. faecalis* SF1** | ***En. faecalis* S1.33** | ***En. faecalis* S1.72b** | ***En. faecalis* S1.63b** |
| --- | --- | --- | --- | --- | --- | --- | --- | --- | --- | --- | --- | --- | --- | --- | --- | --- | --- | --- | --- | --- |
| **E1** | 0 | 0 | 0 | 0 | 0 | 0 | 0 | 0 | 0 | 0 | 0 | 0 | 0 | 0 | 0 | 0 | 0 | 0 | 0 | 0 |
| **E2** | 0 | 0 | 0 | 0 | 0 | 0 | 0 | 0 | 0 | 11 | 0 | 8 | 8 | 0 | 0 | 10 | 9 | 0 | 0 | 0 |
| **E3** | 0 | 0 | 0 | 0 | 0 | 0 | 0 | 0 | 0 | 10 | 11 | 11 | 11 | 9 | 12 | 17 | 15 | 10 | 12 | 11 |
| **E4** | 0 | 11 | 0 | 15 | 12 | 13 | 10 | 10 | 12 | 13 | 13 | 14 | 15 | 12 | 13 | 15 | 14 | 13 | 14 | 12 |
| **E5** | 10 | 12 | 10 | 10 | 8 | 12 | 10 | 10 | 12 | 13 | 15 | 11 | 13 | 15 | 11 | 15 | 15 | 15 | 15 | 15 |
| **E6** | 8 | 8 | 0 | 0 | 8 | 0 | 0 | 0 | 8 | 12 | 8 | 11 | 0 | 12 | 10 | 10 | 10 | 10 | 8 | 0 |
| **E7** | 8 | 0 | 10 | 0 | 10 | 0 | 10 | 8 | 8 | 9 | 0 | 0 | 11 | 0 | 10 | 0 | 10 | 0 | 0 | 11 |
| **E8** | 18 | 10 | 0 | 15 | 14 | 20 | 10 | 10 | 14 | 17 | 17 | 17 | 15 | 16 | 20 | 18 | 18 | 15 | 14 | 16 |
| **E9** | 0 | 8 | 0 | 15 | 13 | 13 | 0 | 0 | 10 | 15 | 15 | 14 | 15 | 15 | 15 | 13 | 20 | 14 | 12 | 14 |
| **M1** | 8 | 8 | 0 | 8 | 0 | 0 | 8 | 8 | 8 | 0 | 0 | 0 | 0 | 9 | 0 | 0 | 0 | 0 | 0 | 0 |
| **M2** | 0 | 0 | 0 | 0 | 0 | 0 | 0 | 0 | 0 | 0 | 0 | 0 | 0 | 0 | 0 | 0 | 0 | 0 | 0 | 0 |
| **M3** | 0 | 0 | 0 | 0 | 0 | 0 | 0 | 0 | 0 | 0 | 0 | 0 | 0 | 0 | 0 | 0 | 0 | 0 | 0 | 0 |
| **M4** | 0 | 8 | 11 | 12 | 8 | 8 | 8 | 8 | 8 | 8 | 8 | 8 | 8 | 8 | 10 | 9 | 10 | 8 | 10 | 0 |
| **M5** | 10 | 10 | 10 | 11 | 8 | 8 | 11 | 12 | 10 | 9 | 10 | 11 | 11 | 11 | 8 | 8 | 8 | 12 | 10 | 11 |
| **M6** | 0 | 0 | 0 | 0 | 0 | 0 | 0 | 0 | 0 | 0 | 0 | 0 | 0 | 0 | 0 | 0 | 0 | 0 | 0 | 0 |
| **M7** | 0 | 0 | 0 | 0 | 0 | 0 | 0 | 0 | 0 | 0 | 0 | 0 | 0 | 0 | 0 | 0 | 0 | 0 | 0 | 0 |
| **M8** | 8 | 8 | 17 | 8 | 10 | 8 | 15 | 12 | 17 | 9 | 14 | 14 | 12 | 14 | 18 | 12 | 12 | 13 | 15 | 10 |
| **M9** | 0 | 8 | 10 | 0 | 0 | 0 | 0 | 0 | 0 | 0 | 8 | 9 | 10 | 11 | 8 | 0 | 0 | 0 | 0 | 0 |
| **I1** | 0 | 0 | 0 | 0 | 0 | 0 | 0 | 0 | 0 | 0 | 0 | 0 | 0 | 0 | 0 | 0 | 0 | 0 | 0 | 0 |
| **I2** | 0 | 0 | 0 | 0 | 8 | 10 | 11 | 10 | 8 | 8 | 10 | 0 | 0 | 0 | 12 | 0 | 8 | 8 | 9 | 10 |
| **I3** | 0 | 0 | 0 | 0 | 0 | 0 | 0 | 0 | 0 | 0 | 0 | 0 | 0 | 0 | 0 | 0 | 0 | 0 | 0 | 0 |
| **I4** | 8 | 0 | 10 | 10 | 0 | 0 | 10 | 10 | 0 | 0 | 0 | 0 | 0 | 0 | 0 | 0 | 0 | 12 | 0 | 0 |
| **I5** | 8 | 8 | 11 | 0 | 8 | 10 | 8 | 8 | 8 | 0 | 0 | 0 | 8 | 10 | 10 | 12 | 8 | 8 | 8 | 0 |
| **I6** | 0 | 8 | 8 | 0 | 0 | 10 | 0 | 0 | 8 | 10 | 10 | 0 | 8 | 8 | 8 | 0 | 0 | 10 | 10 | 0 |
| **I7** | 0 | 0 | 0 | 0 | 0 | 0 | 0 | 0 | 0 | 0 | 0 | 0 | 10 | 0 | 0 | 0 | 8 | 0 | 8 | 8 |
| **I8** | 0 | 0 | 12 | 0 | 0 | 0 | 11 | 0 | 0 | 0 | 10 | 8 | 8 | 8 | 10 | 11 | 0 | 0 | 0 | 0 |
| **I9** | 0 | 8 | 0 | 0 | 0 | 0 | 0 | 0 | 8 | 0 | 0 | 10 | 0 | 0 | 0 | 10 | 8 | 10 | 12 | 10 |

**Table S3:** Antibacterial activity of the plant extracts on the *Lacticaseibacillus* isolates and the three reference strains as determined by the agar disc diffusion assay. The diameters of the inhibition zones (mm) were calculated as the mean of three independent repeats. E: ethanolic extract; I: aqueous extract obtained by infusion; M: aqueous extract obtained by maceration.

|  | ***Lb. rhamnosus* L14** | ***Lb. rhamnosus* L15** | ***Lb. rhamnosus* L16** | ***Lb. rhamnosu*s L21** | ***Lb. rhamnosus* L27** | ***Lb. paracase*i L2.2** | ***Lb. rhamnosus* L2** | ***Lb. rhamnosus* L33** | ***Lb. rhamnosus* L36** | ***Lb. rhamnosus* L41.1** | ***Lb. rhamnosus* L41.2** | ***Lb. rhamnosus* L46** | ***Lb. rhamnosus* L50** | ***Lb. rhamnosus* L51** | ***Lb. paracasei* L6** | ***Lb. rhamnosus* L55** | ***Lb. rhamnosus* L17** | ***Lb. paracase*i L2.22** | ***Lb. paracasei* L2.26** | ***Lb. rhamnosus* L11** | ***Str. mutans* ATCC 25175** | ***C. albicans* ATCC 28366** | ***F. nucleatum* ATCC 25586** |
| --- | --- | --- | --- | --- | --- | --- | --- | --- | --- | --- | --- | --- | --- | --- | --- | --- | --- | --- | --- | --- | --- | --- | --- |
| **E1** | 0 | 0 | 0 | 0 | 0 | 0 | 0 | 0 | 0 | 0 | 0 | 0 | 0 | 0 | 0 | 0 | 0 | 0 | 0 | 0 | 0 | 0 | 9 |
| **E2** | 0 | 0 | 0 | 0 | 0 | 0 | 0 | 0 | 0 | 0 | 0 | 0 | 0 | 0 | 0 | 0 | 0 | 0 | 0 | 0 | 11 | 0 | 12 |
| **E3** | 0 | 0 | 0 | 0 | 0 | 0 | 0 | 0 | 0 | 0 | 0 | 0 | 0 | 0 | 0 | 0 | 0 | 0 | 0 | 0 | 11 | 0 | 8 |
| **E4** | 0 | 13 | 0 | 12 | 0 | 11 | 12 | 11 | 11 | 12 | 11 | 11 | 11 | 10 | 11 | 12 | 10 | 11 | 12 | 11 | 17 | 0 | 18 |
| **E5** | 8 | 11 | 10 | 13 | 11 | 10 | 12 | 14 | 11 | 13 | 12 | 12 | 10 | 10 | 10 | 11 | 12 | 10 | 11 | 10 | 18 | 12 | 12 |
| **E6** | 9 | 0 | 0 | 0 | 0 | 0 | 8 | 8 | 0 | 9 | 9 | 0 | 0 | 0 | 8 | 10 | 0 | 8 | 8 | 8 | 11 | 0 | 10 |
| **E7** | 8 | 0 | 10 | 8 | 0 | 0 | 9 | 10 | 8 | 8 | 9 | 0 | 10 | 8 | 9 | 10 | 7 | 8.5 | 8 | 9 | 11 | 0 | 18 |
| **E8** | 16 | 12 | 0 | 15 | 15 | 13 | 14 | 13 | 13 | 14 | 13 | 12 | 11 | 15 | 0 | 13 | 11 | 14.5 | 12 | 0 | 30 | 34 | 18 |
| **E9** | 0 | 11 | 0 | 14 | 0 | 14 | 10 | 10 | 10 | 11 | 12 | 10 | 10 | 13 | 0 | 10 | 11 | 9 | 7 | 0 | 18 | 0 | 19 |
| **M1** | 8 | 8 | 0 | 11 | 8 | 8 | 10 | 11 | 12 | 11 | 8 | 8 | 8 | 12 | 8 | 0 | 0 | 0 | 0 | 8 | 0 | 0 | 0 |
| **M2** | 0 | 0 | 0 | 0 | 0 | 0 | 0 | 0 | 0 | 0 | 0 | 0 | 0 | 0 | 0 | 0 | 0 | 0 | 0 | 0 | 0 | 0 | 0 |
| **M3** | 0 | 0 | 0 | 0 | 0 | 0 | 0 | 0 | 0 | 0 | 0 | 0 | 0 | 0 | 0 | 0 | 0 | 0 | 0 | 0 | 0 | 0 | 0 |
| **M4** | 8 | 0 | 0 | 10 | 8 | 8 | 0 | 9 | 10 | 12 | 0 | 9 | 14 | 12 | 11 | 10 | 12 | 8 | 0 | 8 | 0 | 0 | 0 |
| **M5** | 8 | 12 | 8 | 8 | 10 | 10 | 11 | 12 | 13 | 8 | 8 | 8 | 8 | 10 | 11 | 11 | 8 | 12 | 12 | 8 | 0 | 0 | 0 |
| **M6** | 0 | 0 | 0 | 0 | 0 | 0 | 0 | 0 | 0 | 0 | 0 | 0 | 0 | 0 | 0 | 0 | 0 | 0 | 0 | 0 | 0 | 0 | 0 |
| **M7** | 8 | 0 | 0 | 10 | 8 | 0 | 0 | 0 | 0 | 0 | 0 | 0 | 0 | 0 | 0 | 0 | 0 | 0 | 0 | 8 | 0 | 0 | 0 |
| **M8** | 0 | 0 | 0 | 0 | 0 | 0 | 0 | 0 | 0 | 0 | 0 | 0 | 0 | 0 | 0 | 0 | 0 | 0 | 0 | 0 | 20 | 12 | 17 |
| **M9** | 0 | 0 | 0 | 0 | 0 | 0 | 0 | 0 | 0 | 0 | 0 | 0 | 0 | 0 | 0 | 0 | 0 | 0 | 0 | 0 | 20 | 0 | 14 |
| **I1** | 0 | 0 | 0 | 0 | 0 | 0 | 0 | 0 | 0 | 0 | 0 | 0 | 0 | 0 | 0 | 0 | 0 | 0 | 0 | 0 | 0 | 0 | 0 |
| **I2** | 0 | 0 | 0 | 0 | 8 | 10 | 11 | 10 | 8 | 8 | 10 | 0 | 0 | 0 | 12 | 0 | 8 | 8 | 9 | 10 | 8 | 0 | 0 |
| **I3** | 0 | 0 | 0 | 0 | 0 | 0 | 0 | 0 | 0 | 0 | 0 | 0 | 0 | 0 | 0 | 0 | 0 | 0 | 0 | 0 | 0 | 0 | 0 |
| **I4** | 8 | 0 | 10 | 10 | 0 | 0 | 10 | 10 | 0 | 0 | 0 | 0 | 0 | 0 | 0 | 0 | 0 | 12 | 0 | 0 | 8 | 0 | 0 |
| **I5** | 8 | 8 | 11 | 0 | 8 | 10 | 8 | 8 | 8 | 0 | 0 | 0 | 8 | 10 | 10 | 12 | 8 | 8 | 8 | 0 | 0 | 0 | 0 |
| **I6** | 0 | 8 | 8 | 0 | 0 | 10 | 0 | 0 | 8 | 10 | 10 | 0 | 8 | 8 | 8 | 0 | 0 | 10 | 10 | 0 | 8 | 0 | 0 |
| **I7** | 0 | 0 | 0 | 0 | 0 | 0 | 0 | 0 | 0 | 0 | 0 | 0 | 10 | 0 | 0 | 0 | 8 | 0 | 8 | 8 | 0 | 0 | 0 |
| **I8** | 0 | 0 | 12 | 0 | 0 | 0 | 11 | 0 | 0 | 0 | 10 | 8 | 8 | 8 | 10 | 11 | 0 | 0 | 0 | 0 | 8 | 10 | 10 |
| **I9** | 0 | 8 | 0 | 0 | 0 | 0 | 0 | 0 | 8 | 0 | 0 | 10 | 0 | 0 | 0 | 10 | 8 | 10 | 12 | 10 | 8 | 8 | 10 |

### Screening the antimicrobial activity of the plant extracts on planktonic cultures

**Table S4:** Planktonic growth inhibition exerted by the plant extracts on the indicated microorganism. Values represent inhibition percentages compared to a negative control and were calculated as the mean of three independent repeats. E: ethanolic extract; I: aqueous extract obtained by infusion; M: aqueous extract obtained by maceration.

|  | ***Str. mitis* SA5** | ***Str. salivarius* SA3** | ***Str. anginosus* S4.49** | ***En. faecalis* S2.43** | ***Str. vestibularis* S2P1** | ***Lb. paracasei* L2.2** | ***Lb. rhamnosus* L41.2** | ***Lb. rhamnosus* L27** | ***Lb. rhamnosus* L46** | ***Lb. paracasei* L6** | ***C. albicans* ATCC 28366** | ***Str. mutans* ATCC 25175** |
| --- | --- | --- | --- | --- | --- | --- | --- | --- | --- | --- | --- | --- |
| **E1** | 40.30 | 54.91 | 40 | 51.43 | 57.48 | 35.65 | 47.13 | 63.22 | 73.85 | 32.64 | 55.87 | 45.32 |
| **E2** | 58.31 | 99.96 | 45 | 52.51 | 99.55 | 12.68 | 34.15 | 55.29 | 24.40 | 37.46 | 87.06 | 35.6 |
| **E3** | 63.86 | 99.16 | 60.32 | 60.76 | 96.99 | 49.81 | 34.59 | 34.21 | 16.66 | 65.63 | 34.70 | 16.8 |
| **E4** | 71.81 | 86.82 | 19.83 | 88.58 | 77.90 | 87.31 | 28.97 | 57.30 | 54.35 | 59.10 | 99.36 | 45.65 |
| **E5** | 99.36 | 99.15 | 89.9 | 95.62 | 99.2 | 50 | 99.59 | 68.39 | 65.64 | 99 | 99.5 | 74.6 |
| **E6** | 83.5 | 35.27 | 33.65 | 34.61 | 65.26 | 49.34 | 78.39 | 42.98 | 52.05 | 65.16 | 45.66 | 36.5 |
| **E7** | 87.69 | 76.14 | 74.63 | 51.38 | 87.65 | 57.35 | 30.09 | 57.23 | 64.69 | 46.67 | 87 | 18.6 |
| **E8** | 79.18 | 86.92 | 90.08 | 89.23 | 89.91 | 88.33 | 89.90 | 64.25 | 69.54 | 73.22 | 89.19 | 77.5 |
| **E9** | 85.38 | 68.93 | 24.55 | 79.58 | 99.24 | 12.80 | 44.30 | 22.90 | 56.18 | 62.87 | 51.97 | 65.64 |
| **M1** | 48.54 | 2.91 | 0.54 | 2.69 | 19.23 | 0.56 | 14.34 | 18.12 | 21.56 | 21.18 | 5.67 | 31.87 |
| **M2** | 38.22 | 15.21 | 0.35 | 26.51 | 12.69 | 0.12 | 0.36 | 8.91 | 24.50 | 32.12 | 47.94 | 49.58 |
| **M3** | 18.79 | 48.41 | 0.22 | 47.69 | 82.54 | 46.55 | 50.91 | 58.47 | 62.57 | 78.55 | 62.14 | 12.85 |
| **M4** | 63.58 | 49.11 | 11.46 | 41.41 | 60.01 | 53.44 | 63.41 | 58.04 | 70.26 | 76.00 | 68.45 | 21.66 |
| **M5** | 91.98 | 50.37 | 82.31 | 0.14 | 49.78 | 30.89 | 60.54 | 67.58 | 62.41 | 62.53 | 29.96 | 28.22 |
| **M6** | 78.70 | 39.27 | 67.12 | 25.61 | 36.10 | 0.01 | 4.64 | 82.86 | 65.57 | 58.78 | 15.14 | 56.85 |
| **M7** | 47.68 | 35.27 | 0.18 | 17.69 | 13.30 | 21.55 | 7.17 | 3.50 | 0.5 | 20.93 | 0.94 | 7.58 |
| **M8** | 76.32 | 61.83 | 52.84 | 67.23 | 64.01 | 44.92 | 53.58 | 74.16 | 57.84 | 0.13 | 68.45 | 5.95 |
| **M9** | 89.76 | 59.06 | 24.39 | 63.15 | 78.48 | 57.97 | 44.30 | 51.75 | 32.78 | 34.68 | 44.16 | 50.36 |
| **I1** | 59.69 | 35.27 | 0.26 | 25.38 | 44.18 | 18.47 | 0.35 | 37.13 | 32.02 | 21.05 | 9.14 | 25.16 |
| **I2** | 75.87 | 79.71 | 11.21 | 61.23 | 36.10 | 78.14 | 24.05 | 81.18 | 69.38 | 70.71 | 51.10 | 13.16 |
| **I3** | 65.00 | 89.10 | 58.37 | 56.71 | 40.14 | 87.62 | 6.46 | 35.18 | 9.38 | 78.12 | 57.09 | 3.65 |
| **I4** | 74.07 | 50.26 | 16.68 | 45.94 | 57.64 | 21.25 | 10.40 | 56.82 | 71.02 | 78.72 | 29.65 | 11.66 |
| **I5** | 37.95 | 45.73 | 21.46 | 49.10 | 75.95 | 53.80 | 44.64 | 50.43 | 67.30 | 57.68 | 29.96 | 36.65 |
| **I6** | 62.26 | 44.44 | 5.60 | 77.69 | 46.43 | 19.74 | 21.09 | 27.09 | 8.00 | 49.52 | 19.55 | 11.64 |
| **I7** | 53.68 | 2.91 | 6.34 | 45.64 | 34.67 | 25.72 | 0.84 | 3.50 | 3.59 | 17.70 | 12.30 | 18.65 |
| **I8** | 61.92 | 59.87 | 1.46 | 59.23 | 48.53 | 3.62 | 34.73 | 67.56 | 18.40 | 45.21 | 22.39 | 36.56 |
| **I9** | 63.97 | 67.63 | 26.82 | 45.64 | 45.36 | 31.52 | 40.08 | 0.33 | 20.91 | 26.95 | 13.24 | 14.65 |

### Screening the anti-biofilm activity of the plant extracts

**Table S5:** Biofilm inhibition exerted by the plant extracts on the indicated microorganism. Values represent inhibition percentages compared to a negative control and were calculated as the mean of three independent repeats. E: ethanolic extract; I: aqueous extract obtained by infusion; M: aqueous extract obtained by maceration.

|  | ***Str. mitis* SA5** | ***Str. salivarius* SA3** | ***Str. anginosus* S4.49** | ***En. faecali*s S2.43** | ***Str. vestibularis* S2P1** | ***Lb. paracasei* L2.2** | ***Lb. rhamnosus* L41.2** | ***Lb. rhamnosus* L27** | ***Lb. rhamnosus* L46** | ***Lb. paracasei* L6** | ***C. albicans* ATCC 28366** | ***Str. mutans* ATCC 25175** |
| --- | --- | --- | --- | --- | --- | --- | --- | --- | --- | --- | --- | --- |
| **E1** | 32.52 | 33.02 | 46.13 | 60.74 | 60.81 | 17.91 | 49.17 | 28.39 | 35.85 | 32.97 | 25.11 | 45.12 |
| **E2** | 40.88 | 99.90 | 15.00 | 26.32 | 86.90 | 29.94 | 30.23 | 52.04 | 52.18 | 14.37 | 56.97 | 33.50 |
| **E3** | 55.66 | 49.20 | 63.67 | 40.74 | 89.85 | 46.60 | 10.01 | 42.18 | 53.68 | 61.02 | 94.42 | 46.60 |
| **E4** | 44.51 | 35.53 | 27.75 | 35.93 | 36.04 | 33.82 | 46.24 | 64.90 | 52.19 | 41.85 | 61.59 | 40.20 |
| **E5** | 90.15 | 99.32 | 91.20 | 99.81 | 88.34 | 99.84 | 99.91 | 94.92 | 86.28 | 89.80 | 98.18 | 46.33 |
| **E6** | 86.19 | 1.89 | 0.50 | 23.46 | 23.60 | 22.00 | 22.63 | 28.30 | 9.98 | 32.21 | 81.55 | 31.15 |
| **E7** | 97.43 | 57.78 | 26.83 | 53.95 | 64.11 | 40.13 | 7.35 | 41.22 | 39.34 | 0.10 | 35.70 | 42.16 |
| **E8** | 65.75 | 81.75 | 75.90 | 82.10 | 52.19 | 71.75 | 80.15 | 43.16 | 88.79 | 47.67 | 97.85 | 48.00 |
| **E9** | 48.32 | 0.31 | 13.00 | 20.86 | 21.01 | 28.96 | 41.19 | 21.09 | 20.39 | 29.72 | 63.52 | 60.90 |
| **M1** | 5.06 | 0.00 | 9.50 | 5.83 | 6.01 | 0.00 | 0.91 | 8.06 | 1.08 | 13.69 | 6.00 | 88.60 |
| **M2** | 81.09 | 85.30 | 2.80 | 60.96 | 61.04 | 0.24 | 55.21 | 21.94 | 74.98 | 13.92 | 56.97 | 90.67 |
| **M3** | 64.45 | 68.00 | 42.50 | 51.93 | 52.01 | 55.91 | 42.70 | 17.55 | 61.46 | 52.54 | 56.52 | 12.12 |
| **M4** | 75.96 | 43.95 | 8.75 | 28.07 | 28.21 | 58.01 | 18.23 | 34.39 | 76.56 | 44.94 | 53.24 | 44.71 |
| **M5** | 34.47 | 5.28 | 29.53 | 49.44 | 49.54 | 13.23 | 24.67 | 65.31 | 31.24 | 41.51 | 55.26 | 12.50 |
| **M6** | 69.97 | 39.15 | 27.00 | 87.11 | 87.13 | 59.39 | 80.03 | 47.00 | 41.79 | 58.64 | 38.63 | 24.82 |
| **M7** | 10.76 | 2.67 | 4.67 | 23.83 | 23.97 | 5.66 | 2.60 | 6.12 | 4.36 | 3.99 | 7.22 | 57.54 |
| **M8** | 62.40 | 49.73 | 55.96 | 53.63 | 33.72 | 45.93 | 37.46 | 24.15 | 32.68 | 20.63 | 54.94 | 89.12 |
| **M9** | 20.38 | 26.65 | 26.63 | 42.47 | 42.58 | 30.22 | 40.62 | 36.53 | 12.88 | 47.02 | 54.94 | 60.13 |
| **I1** | 7.26 | 0.31 | 0.20 | 18.52 | 18.67 | 5.99 | 7.65 | 19.86 | 16.05 | 35.33 | 17.81 | 12.33 |
| **I2** | 78.16 | 55.03 | 66.25 | 66.54 | 66.61 | 24.72 | 31.75 | 73.61 | 60.44 | 20.97 | 39.81 | 45.23 |
| **I3** | 77.04 | 59.62 | 13.33 | 42.10 | 42.21 | 12.38 | 62.76 | 53.74 | 65.12 | 3.56 | 60.09 | 33.21 |
| **I4** | 73.42 | 25.79 | 38.67 | 19.88 | 20.02 | 37.38 | 71.56 | 59.59 | 4.88 | 62.05 | 18.67 | 33.70 |
| **I5** | 1.94 | 51.24 | 49.25 | 5.25 | 5.05 | 74.27 | 72.87 | 68.71 | 64.30 | 72.11 | 38.84 | 31.36 |
| **I6** | 36.42 | 1.19 | 15.32 | 7.41 | 7.58 | 25.73 | 37.82 | 69.93 | 68.69 | 83.78 | 33.91 | 18.60 |
| **I7** | 9.65 | 1.64 | 6.67 | 1.20 | 1.39 | 6.15 | 7.68 | 14.90 | 15.63 | 0.10 | 12.04 | 22.15 |
| **I8** | 57.61 | 25.47 | 33.50 | 17.42 | 17.57 | 50.53 | 32.23 | 34.62 | 65.40 | 37.62 | 61.37 | 45.60 |
| **I9** | 67.93 | 13.52 | 6.00 | 32.06 | 32.19 | 29.45 | 44.39 | 37.01 | 55.61 | 62.27 | 61.37 | 11.90 |

## *In vitro* biocompatibility of the *O. vulgare* ethanolic extract

## In-depth characterization of the anti-biofilm potential of the *O. vulgare* ethanolic extract

**Figure S2.** Antimicrobial potential of the *O. vulgare* ethanolic extract. Inhibitory effect of the extract on the growth of dental plaque isolates after 24h of incubation. Results were calculated as the means ± SD of three independent repeats. The bars with significant differences were indicated with asterisks. *: (P value < 0.1), **: (P value < 0.01), ***: (P value < 0.001), ****: (P value < 0.0001).

### Ability of the *O. vulgare* ethanolic extract to eradicate pre-formed oral biofilms

**Figure S3.** Effect of the *Origanum vulgare* ethanolic extract on 24-h biofilms of the indicated dental plaque isolate grown on a polystyrene microplate. Biofilms were pre-formed in either TSB broth or in artificial saliva and were treated for two hours with the *O. vulgare* extract. Results are presented as bacterial cell counts and were calculated as the means ± SD of three independent repeats. The bars with significant differences from the negative control were indicated with asterisks. *: (*P* value < 0.1), **: (*P* value < 0.01), ***: (*P* value < 0.001), ****: (*P* value < 0.0001).

**Figure S4.** Effect of the *Origanum vulgare* ethanolic extract on 7-day biofilms of the indicated dental plaque isolate grown on a polystyrene microplate. Biofilms were pre-formed in either TSB broth or in artificial saliva and were treated for two hours with the *O. vulgare* extract. Results are presented as bacterial cell counts and were calculated as the means ± SD of three independent repeats. The bars with significant differences from the negative control were indicated with asterisks. *: (*P* value < 0.1), **: (*P* value < 0.01), ***: (*P* value < 0.001), ****: (*P* value < 0.0001).

**Figure S5.** Effect of the *Origanum vulgare* ethanolic extract on 24-h biofilms of the indicated dental plaque isolate grown on a hydroxyapatite-coated microplate. Biofilms were pre-formed in either TSB broth or in artificial saliva and were treated for two hours with the *O. vulgare* extract. Results are presented as a relative fluorescence after incubation with resazurin and were calculated as the means ± SD of three independent repeats. The bars with significant differences from the negative control were indicated with asterisks. *: (*P* value < 0.1), **: (*P* value < 0.01), ***: (*P* value < 0.001), ****: (*P* value < 0.0001).

**Figure S6.** Effect of the *Origanum vulgare* ethanolic extract on 7-day biofilms of the indicated dental plaque isolate grown on a hydroxyapatite-coated microplate. Biofilms were pre-formed in either TSB broth or in artificial saliva and were treated for two hours with the *O. vulgare* extract. Results are presented as a relative fluorescence after incubation with resazurin and were calculated as the means ± SD of three independent repeats. The bars with significant differences from the negative control were indicated with asterisks. *: (*P* value < 0.1), **: (*P* value < 0.01), ***: (*P* value < 0.001), ****: (*P* value < 0.0001).

## Fractionation of the *O. vulgare* ethanolic extract and characterization of the pure compounds

**Table S6.** Antibacterial activity of fractions of the ethanolic extract of *Origanum vulgare* determined via the agar disc diffusion method. The fractions were obtained via column chromatography using increasingly polar eluents: 100 % heptane [Fr1], 50-50 % heptane-ethylacetate [Fr2], 60-40 % dichloromethane-methanol [Fr3], 100 % methanol [Fr4]. Values represent the mean ± SD of three independent replicates.

| **Fraction** | **Fr1** | **Fr2** | | **Fr3** | **Fr4** |
| --- | --- | --- | --- | --- | --- |
| **Weight (mg)** | 2.004 | 1.2604 | | 0.7181 | 16.0093 |
| **Tested strain** |  | | **Inhibition zone diameter (mm)** | | |
| ***En. faecalis* S2.43** | 18 ± 0.1 | 00 ± 00 | | 7 ± 0.2 | 00 ± 00 |
| ***Lb. rhamnosus* L41.2** | 18 ± 0.1 | 15 ± 0.2 | | 16 ± 0.2 | 9 ± 0.1 |
| ***Lb. paracasei* L2.2** | 9 ± 0.4 | 18 ± 0.3 | | 00 ± 00 | 10 ± 0.1 |
| ***Str. mitis* SA5** | 20 ± 0.5 | 9 ± 00 | | 7 ± 00 | 00 ± 00 |
| ***Str. salivarius* SA3** | 17 ± 0.4 | 00 ± 00 | | 9 ± 0.2 | 00 ± 00 |
| ***Str. anginosus* S4.49** | 18 ± 0.2 | 00 ± 00 | | 7 ± 0.2 | 00 ± 00 |
| ***Str. vestibularis* S2P1** | 16 ± 0.5 | 00 ± 00 | | 9 ± 0.3 | 11 ± 00 |
| ***En. durans* S1.41** | 11 ± 00 | 16 ± 0.4 | | 10 ± 00 | 8 ± 0.5 |
| ***En. faecalis* S2.43** | 18 ± 0.1 | 00 ± 00 | | 7 ± 0.2 | 00 ± 00 |
